# Differential Localization and Independent Acquisition of the H3K9me2 and H3K9me3 Chromatin Modifications in the *Caenorhabditis elegans* Adult Germ Line

**DOI:** 10.1371/journal.pgen.1000830

**Published:** 2010-01-22

**Authors:** Jessica B. Bessler, Erik C. Andersen, Anne M. Villeneuve

**Affiliations:** 1 Departments of Developmental Biology and Genetics, Stanford University, Stanford, California, United States of America; 2 Department of Biology, Howard Hughes Medical Institute, Massachusetts Institute of Technology, Cambridge, Massachusetts, United States of America; The University of North Carolina at Chapel Hill, United States of America

## Abstract

Histone methylation is a prominent feature of eukaryotic chromatin that modulates multiple aspects of chromosome function. Methyl modification can occur on several different amino acid residues and in distinct mono-, di-, and tri-methyl states. However, the interplay among these distinct modification states is not well understood. Here we investigate the relationships between dimethyl and trimethyl modifications on lysine 9 of histone H3 (H3K9me2 and H3K9me3) in the adult *Caenorhabditis elegans* germ line. Simultaneous immunofluorescence reveals very different temporal/spatial localization patterns for H3K9me2 and H3K9me3. While H3K9me2 is enriched on unpaired sex chromosomes and undergoes dynamic changes as germ cells progress through meiotic prophase, we demonstrate here that H3K9me3 is not enriched on unpaired sex chromosomes and localizes to all chromosomes in all germ cells in adult hermaphrodites and until the primary spermatocyte stage in males. Moreover, high-copy transgene arrays carrying somatic-cell specific promoters are highly enriched for H3K9me3 (but not H3K9me2) and correlate with DAPI-faint chromatin domains. We further demonstrate that the H3K9me2 and H3K9me3 marks are acquired independently. MET-2, a member of the SETDB histone methyltransferase (HMTase) family, is required for all detectable germline H3K9me2 but is dispensable for H3K9me3 in adult germ cells. Conversely, we show that the HMTase MES-2, an E(z) homolog responsible for H3K27 methylation in adult germ cells, is required for much of the germline H3K9me3 but is dispensable for H3K9me2. Phenotypic analysis of *met-2* mutants indicates that MET-2 is nonessential for fertility but inhibits ectopic germ cell proliferation and contributes to the fidelity of chromosome inheritance. Our demonstration of the differential localization and independent acquisition of H3K9me2 and H3K9me3 implies that the trimethyl modification of H3K9 is not built upon the dimethyl modification in this context. Further, these and other data support a model in which these two modifications function independently in adult *C. elegans* germ cells.

## Author Summary

Histone methylation is a widespread feature of eukaryotic chromatin and has been implicated in numerous aspects of chromosome function. Methyl marks have been noted to occur on numerous different amino acid residues and in distinct mono-, di-, and tri-methyl states. However, the interplay among these distinct modification states is not well understood. In this work we investigate the relationships between the dimethyl and trimethyl modifications on lysine 9 of histone H3 (H3K9me2 and H3K9me3). Our analysis exploits organizational features of the *C. elegans* germ line that facilitate cytological visualization of distinct chromosomal features and dynamic changes in localization that are associated with different chromatin marks. Despite the fact that H3K9me2 and H3K9me3 modify the same amino acid residue, our work reveals that these marks exhibit very different localization patterns in the adult *C. elegans* germ line and become concentrated on chromatin with different properties. Moreover, we show that these marks are acquired independently, requiring different histone methyltransferases, implying that H3K9me3 is not built upon the H3K9me2 mark. Collectively, our data indicate that H3K9me2 and H3K9me3 are highly autonomous chromatin modifications, functioning independently of one another in the *C. elegans* germ line.

## Introduction

Chromatin methylation is a complex and dynamic feature of eukaryotic chromosomes. Methylation can occur on numerous different sites on the histone H3 and H4 subunits. At some amino acid residues, such as lysine 4 and lysine 36 of histone H3, the presence of methyl modifications is correlated with active gene expression (reviewed in [1]). In contrast, methyl modifications at other residues, including lysine 9 and lysine 27 of histone H3 are often, but not always, correlated with heterochromatin and gene silencing [1]–[4]. The diversity of methyl modifications is partially reflected in the large number of histone methyltransferases (HMTases) encoded by genomes. Further, in addition to modifying a diverse range of histone residues, methyl marks occur in distinct mono-, di- and tri- methyl states. In some instances, histones bearing the di- and tri- methyl modifications at a given amino acid residue exhibit similar genomic distributions (*e.g.* H3K9 methylation in *S. pombe* [5]), or are dependent on the same HMTase (*e.g.* H3K27me2 and H3K27me3 dependence on MES-2 in *C. elegans* germ cells [6]). However, in many cases the relationships between the distinct methylation states and the HMTases that are responsible for generating them are poorly understood.

The *C. elegans* germ line is an excellent system to investigate the dynamic nature of chromatin modifications and roles of histone methyltransferases (HMTases) in establishing and/or maintaining chromatin marks, as several features of this system facilitate the cytological analysis of chromosome dynamics. Germ cells are organized in a temporal/spatial gradient along the length of the gonad, making it possible to simultaneously visualize mitotically proliferating germ cell nuclei and nuclei progressing through all stages of meiotic prophase. Mitotically cycling germ cell nuclei are found at the distal end of the gonad, and as nuclei move proximally they enter meiotic prophase. Upon meiotic entry, chromosomes partially condense and reorganize spatially, and homologous chromosomes pair and synapse. Completion of pairing and synapsis defines entry into the pachytene stage, during which crossover recombination events are completed between the DNA molecules of the aligned chromosome pairs. During the diplotene and diakinesis stages, chromosomes desynapse and further condense resulting in the formation of compact bivalents in which the homolog pairs are held together by chiasmata, the cytological correlates of crossovers [7]. During oocyte meiosis, individual bivalents and chiasmata are readily visible owing to the large nuclear volume of developing oocytes, whereas during spermatogenesis, late prophase bivalents occupy a small volume within the nuclei of primary spermatocytes [8]. Some chromatin modifications exhibit dramatic and highly dynamic localization changes in parallel with the reorganization and remodeling of chromosomes that occurs during meiotic prophase progression [9]. For example, levels of chromosome-associated H3K9me2 increase substantially during progression through the pachytene stage in hermaphrodite germ lines, then drop precipitously following the diplotene stage.

In addition to allowing us to examine large-scale histone modification dynamics as germ cells progress through the stages of meiotic prophase, the *C. elegans* system also permits a more detailed dissection of the genomic distribution of individual marks; the partially condensed state of chromosomes during meiotic prophase makes it possible to identify which individual chromosomes or chromosome regions are associated with distinct chromatin marks. For example, cytological analysis has demonstrated that the autosomes are enriched for H3K36me2 and H3K4me2, while these modifications are underrepresented on the sex chromosomes in both hermaphrodite (XX) and male animals (XO), correlating with the fact that there is very little expression of X-linked genes in the germ line in either sex [6], [9]–[12]. In contrast, both H3K27me2 and H3K27me3 are broadly distributed on all chromosomes, with H3K27me3 enriched on the weakly transcribed X chromosomes [6]. Moreover, in addition to exhibiting a dynamic localization in the hermaphrodite germ line, H3K9me2 is also highly enriched on the partnerless X chromosome in XO males as well as on the unpaired X chromosomes present in a *him-8* mutant hermaphrodite [9],[13]. In addition to facilitating the visualization of endogenous chromosome chromatin, this system also allows us to analyze the chromatin modifications associated with transgene arrays, which are tools commonly used in *C. elegans* transcription studies [9],[14]. These chromosomally-integrated or extrachromosomal elements are of particular interest, as they can exhibit unique chromatin characteristics and have been associated with gene silencing mechanisms that operate both in *cis* and in *trans* [15]–[17]. Notably, a high-copy array that is subjected to germline silencing is heavily dimethylated on lysine 9 in both the hermaphrodite and male germ lines [9].

Several HMTases have been shown to be required for normal germline function in *C. elegans*. MES-2, the *C. elegans* E(z) homolog, is required for H3K27me2 and H3K27me3, in both the embryonic and adult germ line [6]. *mes-2* mutants exhibit a maternal-effect sterile phenotype: homozygous mutants derived from heterozygous mothers are fertile, but the germ lines of their progeny fail to develop properly, resulting in sterility [18]. Loss of MES-4, a different HMTase responsible for H3K36me2 in the germ line, causes a similar maternal-effect sterile phenotype [11],[18]. In contrast, it is currently unknown which histone methyltransferase is required for the dynamic H3K9me2 modification. Additionally, while we do know the identity of a demethylase that can remove H3K9me3 [19], we do not know the identity of the HMTase required for this modification, nor do we have a full understanding of the localization pattern of H3K9me3.

In this work, we have investigated the acquisition and localization of the dimethyl and trimethyl modifications of lysine 9 on histone H3 in the *C. elegans* adult germ line. We find that H3K9me2 and H3K9me3 exhibit very different localization patterns on endogenous chromosomes and on integrated and extrachromosomal transgene arrays. Moreover, we show that these modifications are dependent on distinct HMTases. We identify the SETDB1 homolog, MET-2, as the HMTase required for H3K9me2, while H3K9me3 is partially dependent upon the E(z) homolog, MES-2. These and other data indicate that, in the *C. elegans* adult germ line, H3K9me2 and H3K9me3 are highly autonomous chromatin modifications, localizing and functioning independently of one another.

## Results

### H3K9me2 and H3K9me3 Exhibit Distinct Localization Patterns on Germline Chromosomes

To investigate relationships between the H3K9me2 and H3K9me3 modifications, we used immunofluorescence (IF) and high-resolution imaging to compare the localization of these chromatin marks on chromosomes in the germ cells of adult worms. This analysis revealed very different distributions for H3K9me2 and H3K9me3 in all germ cell stages represented in the germ line.

**Figure 1:**
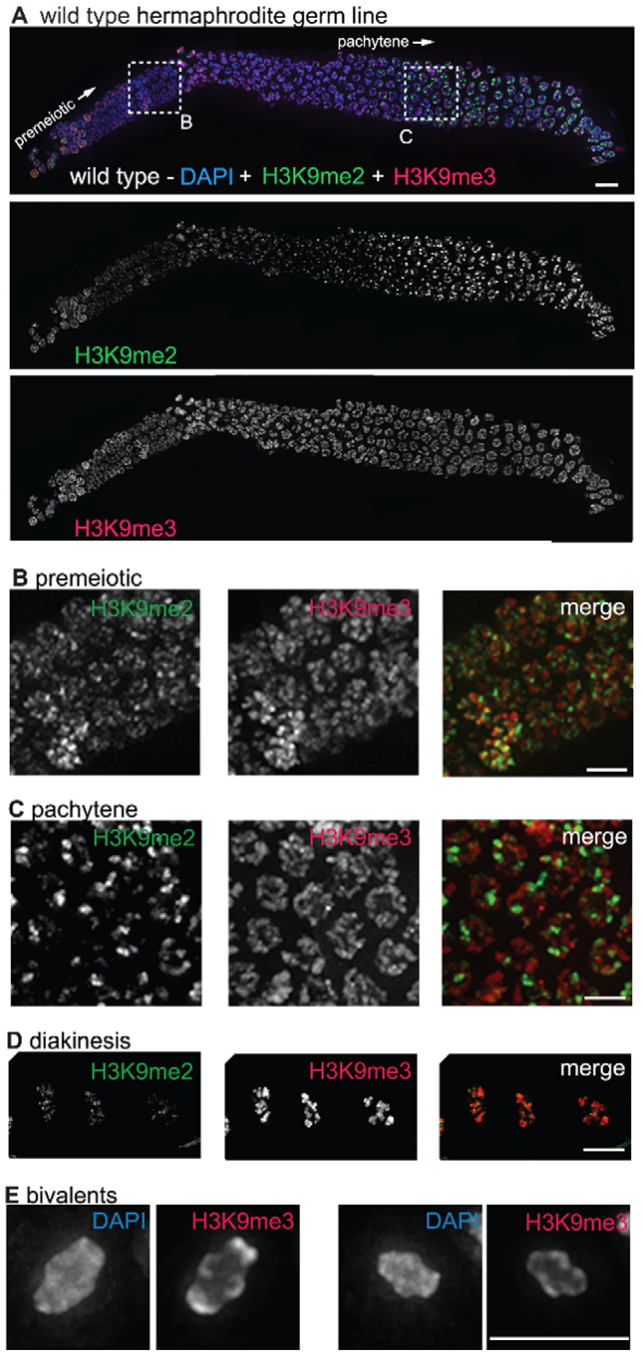
H3K9me2 and H3K9me3 localization in the adult hermaphrodite germ line. (A) A germ line dissected from a wild type *C. elegans* hermaphrodite. The premeiotic region of the germ line is on the left, while germ cells at the diplotene stage of meiotic prophase are on the right. The top panel shows anti-H3K9me2 (green) and anti-H3K9me3 (red) staining overlaid on DAPI-stained chromosomes (blue). The middle panel shows anti-H3K9me2 staining alone and the bottom panel shows anti-H3K9me3 staining alone. Boxed regions are enlarged in sections (B,C). Scale bar = 10 µm. (B) Enlargement of premeiotic nuclei from A, showing that both H3K9me2 and H3K9me3 are broadly distributed, but that their peaks of intensity often do not overlap. Scale bar = 5 µm. (C) Enlargement of pachytene nuclei from A, showing that H3K9me2 is concentrated on a subset of chromosome regions, whereas H3K9me3 is more broadly distributed throughout the chromatin. Scale bar = 5 µm. (D) Examples of wild type diakinesis nuclei, in which H3K9me3 is strongly present on all chromosomes but H3K9me2 is barely detectable. Scale bar = 5 µm. (E) Examples of diakinesis bivalents stained with DAPI and H3K9me3, showing that H3K9me3 exhibits a distinct banding pattern. Scale bar = 5 µm.

H3K9me2 was visualized using a mouse monoclonal antibody (Abcam, ab1220). The dynamic pattern of H3K9me2 staining that we observed during meiotic prophase progression in adult hermaphrodite germ lines closely recapitulated that described in previous reports using a polyclonal antibody [9]. Specifically, in germ cells at the early pachytene stage of meiotic prophase, H3K9me2 was present at very low levels on most of the chromatin and was detected predominantly in a few bright patches per nucleus. The number and size of chromosomal regions harboring bright H3K9me2 staining increased as germ cells progressed through the pachytene stage and continued to increase through the diplotene stage. As germ cells progressed from diplotene to diakinesis, the H3K9me2 modification was lost (Figure 1 and [9]). In addition to the meiotic prophase staining, we also saw anti-H3K9me2 staining in nuclei in more distal regions of the germ line, where H3K9me2 had not been detected in previous studies using rabbit H3K9me2 antibodies (Figure 1A and 1B).

In contrast to the dynamic localization pattern along the gradient of the hermaphrodite germ line observed for H3K9me2, we found that H3K9me3 was present on all chromosomes in all germ cells throughout the gonad, from the mitotic proliferative region through the diakinesis-stage oocytes at the proximal end of the germ line (Figure 1A–1D). (See Materials and Methods for a discussion of different H3K9me3 antibodies utilized.) While H3K9me3 was largely present wherever DAPI-stained chromatin was visible, variations in antibody staining intensity were seen across the chromosomes, most prominently at diakinesis, where distinctive banding patterns could be observed (Figure 1D and 1E). Further, while H3K9me3 staining often did overlap with H3K9me2 staining, the peaks of these marks rarely coincided (Figure 1).

**Figure 2:**
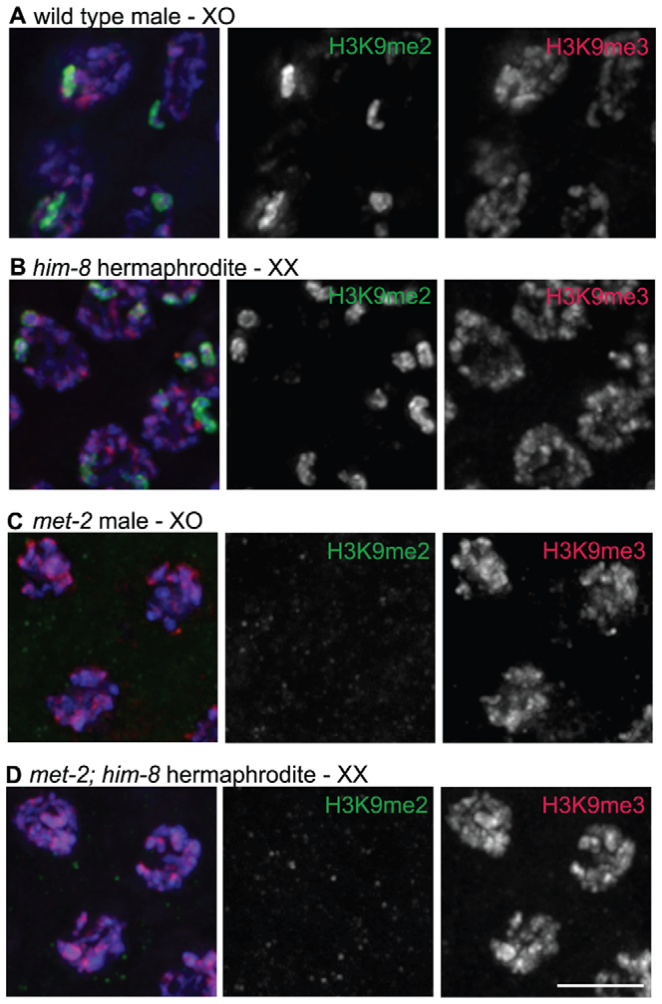
H3K9me2 and H3K9me3 localization in pachytene nuclei from XO males and *him-8* XX hermaphrodites. (A) Male pachytene germ cell nuclei each have a single X chromosome. H3K9me2 is highly enriched on this unpaired X chromosome, while H3K9me3 is present on all chromosomes and is not enriched on the X chromosome. (B) *him-8* hermaphrodite pachytene germ cell nuclei, which carry two unpaired X chromosomes. H3K9me2 is highly enriched on both unpaired X chromosomes, while H3K9me3 is present on all chromosomes. (C) *met-2(n4256)* male pachytene germ cell nuclei. In the *met-2* mutant nuclei, H3K9me2 is no longer present on the unpaired male X chromosome, while the distribution of H3K9me3 appears unaffected. (D) *met-2(n4256)*; *him-8* hermaphrodite pachytene germ cell nuclei. In these nuclei, H3K9me2 is no longer present on the unpaired X chromosomes. The distribution of H3K9me3 appears unaffected. All germs cells have been co-stained with anti-H3K9me2 (green), anti-H3K9me3 (red) and DAPI (blue). Scale bar = 5 µm.

The H3K9me2 and H3K9me3 chromatin modifications also exhibited distinct localization patterns in male germ cell nuclei. In agreement with previous reports, our immunofluorescence analysis using the monoclonal antibody ab1220 detected increasing accumulation of H3K9me2 in male meiotic germ cells beginning at early-pachytene, followed by a loss of detection of H3K9me2 as the germ cells became primary spermatocytes. In males, this accumulation of H3K9me2 occurred mostly on the unpaired single X chromosome, where H3K9me2 became highly concentrated (Figure 2A and [9]). In contrast, H3K9me3 was detected on all chromosomes in nuclei from the proliferative zone through the formation of primary spermatocytes and was not enriched on the single male X chromosome (Figure 2A).

The X chromosomes in *him-8* mutant hermaphrodites fail to pair and synapse and have also been shown to accumulate H3K9me2 [13]. Therefore, we examined the localization of H3K9me2 and H3K9me3 in *him-8* mutant germ lines. Similar to the male X chromosome, we found that while H3K9me2 was highly enriched on the unpaired X chromosomes (Figure 2B and [13]), these chromosomes were not enriched for H3K9me3 (Figure 2B).

### H3K9me3 Is Highly Concentrated on High-Copy Transgene Arrays

**Figure 3:**
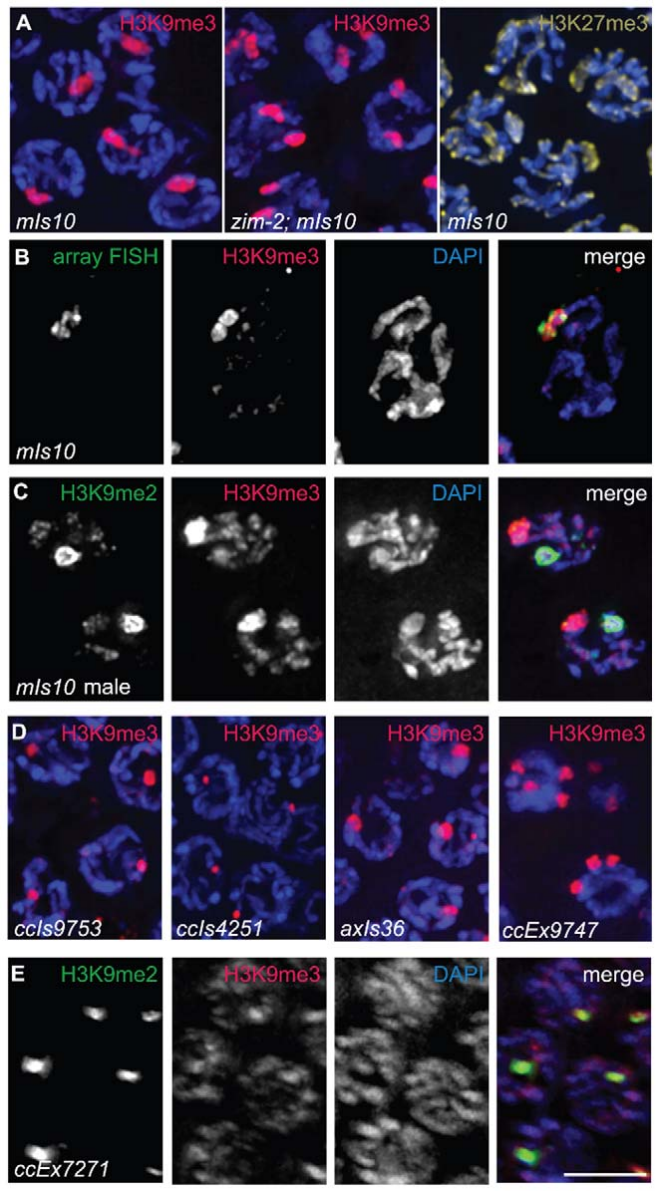
Arrays composed of transgenes with soma-specific promoters are enriched for H3K9me3. (A) Left: Hermaphrodite pachytene nuclei carrying *mIs10*. The *mIs10* array is highly enriched for H3K9me3 (red). Middle: *zim-2*; *mIs10* hermaphrodite pachytene nuclei stained with anti-H3K9me3. *mIs10* is still enriched for H3K9me3 in the *zim-2* mutant despite a lack of pairing between the two copies of chromosome V (into which *mIs10* is integrated). The basal level of H3K9me3 is not visible in these images due to the extreme brightness of H3K9me3-staining associated with *mIs10*. Right: *mIs10* hermaphrodite pachytene nuclei stained for H3K27me3 (yellow). H3K27me3 is broadly distributed on all chromosomes, with a slight enrichment on the X chromosome; it is not concentrated on *mIs10*. Blue = DAPI. (B) *mIs10* hermaphrodite pachytene nucleus hybridized with a high-copy array FISH probe (green), concurrent with anti-H3K9me3 IF (red). The brightest part of the H3K9me3 staining overlaps with the FISH signal. (C) *mIs10* male germ cells co-stained for H3K9me2 (green) and H3K9me3 (red). H3K9me3 is enriched on *mIs10*, while H3K9me2 is enriched on the partnerless X chromosome. (D) Germ cell nuclei carrying different high-copy arrays, composed of soma-specific promoters, stained for H3K9me3 (red). All examined arrays with soma-specific promoters showed an enrichment for H3K9me3. (E) Nuclei containing the high-copy *let-858::gfp* transgene array *ccEx7271*, co-stained for H3K9me2 (green) and H3K9me3 (red). *let-858::gfp* is expressed in the soma but is silenced in the germ line. This array is enriched for H3K9me2, but H3K9me3 levels are indistinguishable from the surrounding chromosomes. Scale bar = 5 µm.

During the course of these experiments, we discovered that germ cell nuclei from worms carrying high-copy transgene arrays exhibited regions of highly elevated anti-H3K9me3 staining. We first made this observation when we detected chromosomal regions highly enriched for H3K9me3 in a strain carrying the *mIs10* array (Figure 3A). *mIs10* carries high-copy transgenes containing promoters that normally function in several different somatic tissues [*myo-2::gfp*; *pes-10::gfp*; *F22B7.9::gfp*], integrated into chromosome V (Edgeley, Riddle, Fire, and Liu pers. comm.). *mIs10*-containing premeiotic nuclei exhibited two enriched anti-H3K9me3 signals, consistent with the unpaired status of chromosomes in this region, while most meiotic prophase nuclei contained one enriched anti-H3K9me3 signal, corresponding to paired chromosomes. Additionally, we examined *zim-2*; *mIs10* mutant worms, in which the chromosome Vs are unpaired [20] (Figure 3A). Consistent with the enrichment of H3K9me3 being due to the presence of *mIs10*, we observed two separate bright anti-H3K9me3 spots in meiotic germ cells from *zim-2*; *mIs10* mutant worms, corresponding to the unpaired copies of *mIs10* on each chromosome V. This result also indicates that the concentration of H3K9me3 on the array during meiosis does not require chromosomes to be paired and synapsed.

To directly confirm that the area of H3K9me3 enrichment was indeed *mIs10*, we developed a Fluorescence *In Situ* Hybridization (FISH) probe to numerous sequences specific to high-copy arrays. Using this FISH probe, in combination with H3K9me3 immunofluorescence, we demonstrated that the *mIs10* array FISH signal and the brightest part of the H3K9me3 IF signal co-localize, which confirmed that the site of H3K9me3 enrichment does correspond to the high-copy array (Figure 3B). Moreover, this analysis also demonstrated that the brightest H3K9me3 staining is largely confined to the array region and does not significantly spread into the neighboring chromatin.

As *mIs10* is associated with an enrichment of H3K9me3, which is generally considered to be a mark of repressive chromatin, we tested whether other repressive modifications might be concentrated on this high-copy transgene array. We examined the localization of H3K27me3, which was previously shown to be enriched on X chromosomes in the germ cells of *C. elegans* hermaphrodites [6]; however, we saw no evidence for the enrichment of H3K27me3 on *mIs10* Figure 3A). Further, as *mIs10* is enriched for H3K9me3 but not H3K27me3, it is highly unlikely that the anti-H3K9me3 signal in the *C. elegans* germ line is due to cross-reactivity with H3K27me3.

We also tested whether H3K9me2 was enriched on *mIs10*. Since H3K9me2 accumulates broadly over multiple different chromosome regions within each nucleus, it was difficult to assess the relationship of H3K9me2 to *mIs10* in hermaphrodite germ cell nuclei. Therefore, we evaluated the relationship of H3K9me2 to *mIs10* in male germ lines where H3K9me2 acquisition is concentrated mainly on the single X chromosome. When male germ cells carrying *mIs10* were co-stained for H3K9me2 and H3K9me3, we detected a single area of strong H3K9me2 enrichment corresponding to the X chromosome, while H3K9me3 exhibited a separate distinct domain of enrichment, reflecting the presence of *mIs10* on chromosome V (Figure 3C). Therefore, of the repressive histone modifications tested, the *mIs10* high-copy arrays only showed an enrichment of H3K9me3.

Examination of several other high-copy transgene arrays (Table 1) indicated that enrichment for H3K9me3 is not restricted to *mIs10*, but is a common feature of high-copy arrays composed of somatically-expressed transgenes. For example, we examined *ccIs9753* [*myo-2::gfp*; *pes-10::gfp*; *F22B7.9::gfp*], which is derived from the same parental extrachromosomal array as *mIs10* but is integrated into chromosome I (Edgeley, Riddle, Fire, and Liu pers. comm.). As nuclei containing *ccIs9753* also had a bright focus of H3K9me3 staining, we can conclude that the enrichment of H3K9me3 on high-copy arrays is not restricted to chromosome V (Figure 3D). Further, the size of the H3K9me3-enriched area in *ccIs9753*-containing nuclei is smaller than that associated with *mIs10*, consistent with suggestions that *ccIs9753* may be a smaller array (Edgeley, Riddle, Fire, and Liu pers. comm.). We also found that *ccIs4251* [*myo-3::Ngfp-lacZ myo-3::Mgfp*] [21], integrated into chromosome I, was enriched for H3K9me3 (Figure 3D), indicating that the presence of high-levels of H3K9me3 is not restricted to arrays carrying the *myo-2*, *pes-10* and *F22B7.9* transgenes.

**Table 1:** High-copy arrays used in this study.

array name	chromosome	transgenes present	promoter type
*mIs10*	V	*myo-2::gfp*	somatic
		*pes-10::gfp*	
		*F22B7.9::gfp*	
*ccIs9753*	I	same as *mIs10*	somatic
*ccEx9747*	extrachromosomal	same as *mIs10*	somatic
*ccIs4251*	I	*myo-3::gfp*	somatic
*axIs36*	X	*pes-10::gfp*	somatic
*ccEx7271*	extrachromosomal	*let-858::gfp*	germline and somatic

We also examined the high-copy array *axIs36* [*pes-10::gfp*], which is integrated into the X chromosome [22]. Although the chromatin of X chromosomes differs from that of autosomes in several ways, including but not limited to an enrichment for the repressive H3K27me3 modification, we nevertheless detected a broad, bright region of H3K9me3 enrichment in nuclei containing *axIs36*. Thus, insertion of a high-copy array into the X chromosome does not preclude recruitment of H3K9me3 to the array (Figure 3D).

The observed enrichment of H3K9me3 on high-copy transgene arrays was not restricted to integrated arrays. We also detected H3K9me3 enrichment on the extrachromosomal array *ccEx9747* [*myo-2::gfp*; *pes-10::gfp*; *F22B7.9::gfp*], the parental DNA from which *mIs10* and *ccIs9753* are derived (Edgeley, Riddle, Fire, and Liu pers. comm.). Further, we were also able to detect varying numbers of extrachromosomal bodies in different germ cells (Figure 3D).

In addition to the above-described high-copy transgene arrays, all of which are composed of transgenes with somatic-cell-specific promoters, we also examined worms containing *ccEx7271*, which is an extrachromosomal array composed of multiple copies of a *let-858::gfp* transgene. The *let-858::gfp* transgene array is distinct because it is subject to germline silencing: while the endogenous *let-858* gene is expressed in both the soma and germ line, *ccEx7271* expresses GFP in the soma but is silenced in the germ line [15]. *ccEx7271* was previously detected as a single bright focus of H3K9me2 staining in each adult germ cell nucleus, and this result was recapitulated by our immunostaining with monoclonal antibody ab1220 (Figure 3E and [9]). However, unlike the other high-copy transgene arrays examined, *ccEx7271* was not highly enriched for H3K9me3. This observation raises the possibility that the presence or absence of germline-competent promoters (albeit silenced) in a transgene array may play a role in defining the chromatin composition of a high-copy array.

### Distinct Properties of H3K9me2 and H3K9me3-Enriched Chromatin Domains

H3K9me2 and H3K9me3 exhibit localization patterns that, while not mutually exclusive, are highly divergent. As H3K9me2 is highly enriched on unpaired X chromosomes, the presence of the heavily trimethylated *axIs36* array integrated into X chromosomes provided an opportunity to investigate the potential interplay between the H3K9me2 and H3K9me3 marks and to identify distinctive features of the chromosome domains to which they become concentrated.

**Figure 4:**
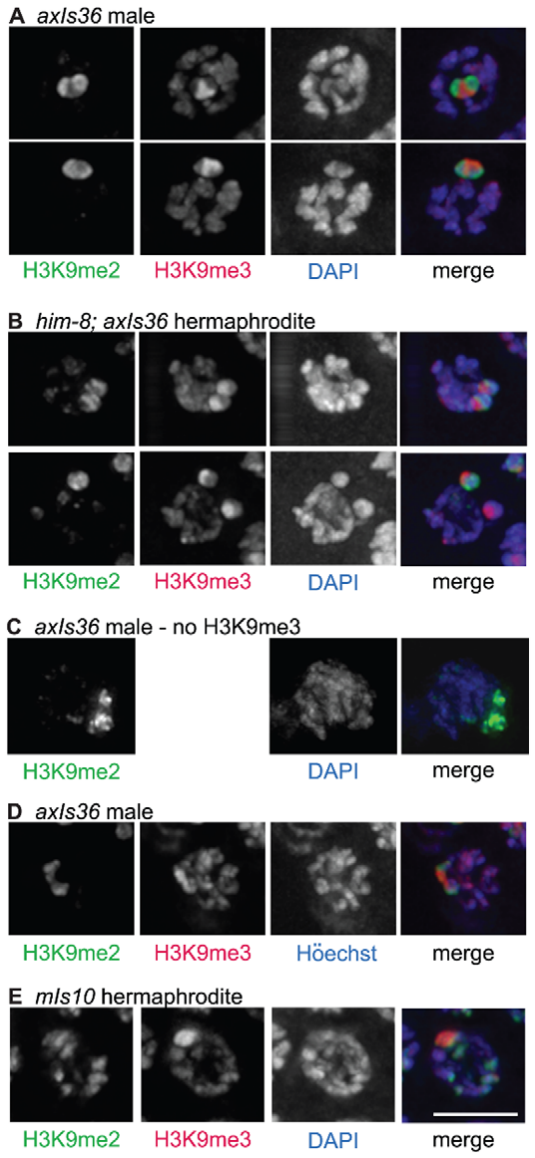
Distinct properties of H3K9me2 and H3K9me3-enriched domains. (A) *axIs36* male pachytene nuclei co-stained with anti-H3K9me2, anti-H3K9me3 and DAPI. H3K9me2 remains restricted to the X chromosome, while H3K9me3 is restricted to *axIs36*. (B) *him-8*; *axIs36* hermaphrodite pachytene nuclei co-stained with anti-H3K9me2, anti-H3K9me3 and DAPI. Top: a nucleus where both X chromosomes carrying *axIs36* stain for H3K9me2 and H3K9me3. 11 of 20 nuclei examined showed two distinct H3K9me2 signals and two distinct H3K9me3 signals. Bottom: a nucleus where one X chromosome carrying *axIs36* is stained only for H3K9me3 and the other X chromosome is stained for both H3K9me2 and H3K9me3. 5 of 20 nuclei examined showed two distinct H3K9me3 signals but only one H3K9me2 signal (see text for additional information). In both (A,B), H3K9me3 staining also corresponds to the presence of a DAPI-faint region. (C) *axIs36* male nucleus stained only with anti-H3K9me2 and DAPI. A gap in the H3K9me2 staining indicates the position of the *axIs36* transgene array inserted into the X chromosome and also corresponds to a region of reduced DAPI intensity, demonstrating that a DAPI-faint region corresponding to *axIs36* is still observed in experiments that do not include anti-H3K9me3 antibody. (D) *axIs36* male nucleus co-stained with anti-H3K9me2, anti-H3K9me3 and Höechst dye #33258. These data indicate that faint chromatin staining coincident with *axIs36* can be seen using Höechst as well as DAPI. (E) *mIs10* hermaphrodite nucleus co-stained for anti-H3K9me2, anti-H3K9me3 and DAPI. A DAPI-faint domain corresponds to the presence of the *mIs10* high-copy array. Green = H3K9me2, Red = H3K9me3, Blue = DAPI or Höechst. Scale bar = 5 µm.

Simultaneous immunolocalization of H3K9me2 and H3K9me3 in male germ cells carrying *axIs36* revealed a clear distinction between chromosomal domains where these marks are concentrated. Specifically, in pachytene nuclei, we detected a region of heavy H3K9me3 staining, correlating to the presence of *axIs36*. This trimethylated region was surrounded by strong anti-H3K9me2 staining, corresponding to the chromatin of the native X chromosome (Figure 4A). Therefore, despite its location on an unpaired X chromosome, *axIs36* did not recruit high levels of H3K9me2. Conversely, the presence of *axIs36* did not interfere with the acquisition of H3K9me2 on the host X chromosome. These findings emphasize the very different character of the chromatin domains carrying these two marks. Further, these results indicate that the state of being unpaired is not sufficient for a chromosomal domain to acquire H3K9me2 in meiosis.

We also examined the germ lines of *him-8* mutant hermaphrodites carrying *axIs36*. We observed that 55% of nuclei examined (11 of 20) contained two well-separated H3K9me2-enriched domains, corresponding to unpaired X chromosomes. On each of the X chromosomes in these 11 nuclei we could also differentiate a spatially distinct domain enriched for H3K9me3 (Figure 4B, top), consistent with results from the XO male germ line. However, in control *him-8* mutant hermaphrodites without *axIs36*, two H3K9me2 enriched domains were visible more frequently, in 85% (55 of 65) of nuclei examined (p<0.01), suggesting either that pairing of X chromosomes was improved or that H3K9me2 accumulation on unpaired X chromosomes was inhibited by the presence of *axIs36*. Therefore, we more closely examined the nine *him-8*; *axIs36* nuclei in which only one H3K9me2-enriched territory could be distinguished. In four of these nine nuclei, the close proximity between H3K9me3-enriched domains suggested that the two X chromosomes were likely to be too close to one another to be resolved as separate territories, as is likely the case for the 15% of *him-8* nuclei with only one discernable H3K9me2-enriched region. However, in the remaining five nuclei, two well-separated regions of H3K9me3 staining were detected, indicating that the two X chromosomes were clearly unpaired (Figure 4B, bottom). Thus, in 5 of 16 nuclei in which the X chromosomes were demonstrably unpaired, one of the two X chromosomes had failed to acquire H3K9me2. These observations raise the possibility that the presence of H3K9me3, or some other feature of the transgene array, may have the capacity to interfere with the ability of female germ cells to sense the presence of an unpaired chromosome and/or to concentrate H3K9me2 in response, possibly by enabling autosynapsis of an unpaired chromosome.

A closer examination of the imaged nuclei in the aforementioned experiments revealed that the H3K9me3-enriched regions corresponded to chromatin with a relatively weak DAPI signal (DAPI-faint), suggestive of a more open chromatin organization (Figure 4A and 4B). These DAPI- faint regions corresponding to *axIs36* were also detectable when only H3K9me2 staining was used to demarcate the location of the X chromosome (Figure 4C), indicating that the presence of this feature was not due to the use of the H3K9me3 antibody. Further, the distinctive appearance of the *axIs36* chromatin domain was not dependent upon use of DAPI, as similar results were obtained using Höechst 33258 (Figure 4D). Finally, a DAPI-faint chromatin domain was observed not only for a high-copy array integrated into the X-chromosome, but also for the *mIs10* array integrated into chromosome V (Figure 4E).

Association of concentrated H3K9me3 signals with DAPI-faint chromatin domains was surprising, as H3K9me3 has been reported to be associated with classical DAPI-bright heterochromatin regions in mammalian cells [23]. Our finding that H3K9me3 associates with DAPI-faint regions indicates either that H3K9me3 does not demarcate heterochromatin in the *C. elegans* germ line or that *C. elegans* may lack heterochromatin consistent with the classical definition, as has been previously suggested [24].

### MET-2 Is Required for H3K9me2 but Not H3K9me3 on Germline Chromatin

We identified MET-2 as a candidate histone methyltransferase (HMTase) responsible for germline methylation on H3K9 based on several criteria. First, MET-2 is homologous to the SETDB1/dSETDB1/ESET family of histone methyltransferases, which are known to methylate histone H3 on lysine 9 *in vitro* [25]–[28]. Second, MET-2 is required to maintain germline immortality, suggesting a function in the germ line [29]. Third, a MudPit mass spectrometry analysis identified MET-2 in immunoprecipitates of GFP-tagged HIM-17 (JBB, AMV, I. McLeod and J. Yates, unpublished), a protein previously demonstrated to be required for the normal patterning of H3K9me2 in the *C. elegans* germ line [30].

**Figure 5:**
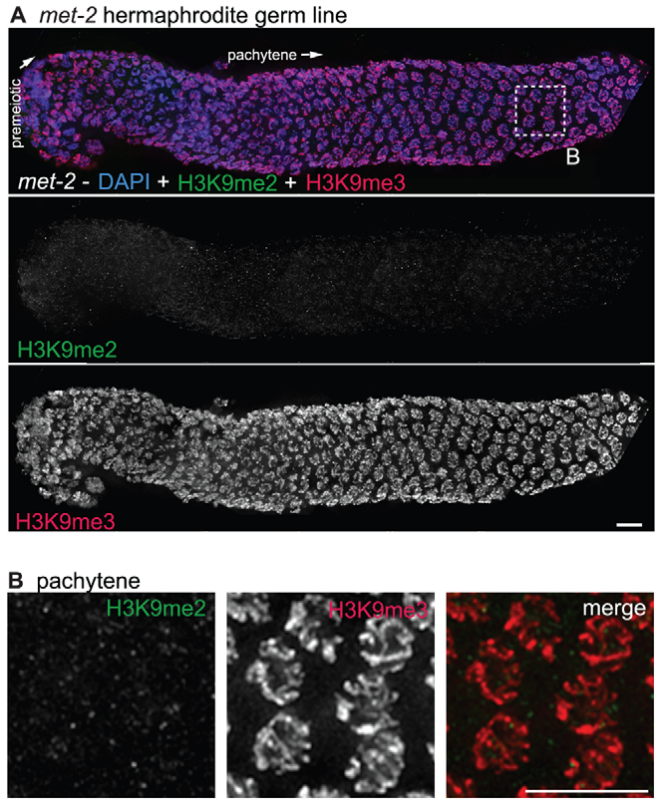
*met-2* is required for H3K9me2 in the adult germ line. (A) A germ line dissected from a *met-2(n4256) C. elegans* hermaphrodite. The premeiotic region of the germ line is on the left, while germ cells at the diplotene stage of meiotic prophase are on the right. Top: anti-H3K9me2 staining (green) and anti-H3K9me3 staining (red) overlaid on DAPI-stained chromosomes (blue). Middle: anti-H3K9me2 staining alone. Bottom: anti-H3K9me3 staining alone. In the absence of MET-2, no chromosomal H3K9me2 staining is visible. Boxed region is enlarged in (B). Scale bar = 10 µm. (B) Enlargement of pachytene nuclei from A, showing that H3K9me2 is not visible in the *met-2(n4256)* mutant germ line, while H3K9me3 is unaffected. Scale bar = 10 µm.

To determine if MET-2 is required for the acquisition or maintenance of H3K9me2 or H3K9me3 in adult *C. elegans* germ cell nuclei, we analyzed germ lines from worms homozygous for either of two independently-derived *met-2* loss-of-function alleles, *met-2(n4256)* [29] or *met-2(ok2307)*. In hermaphrodites homozygous for either *met-2* mutant allele, no germline H3K9me2 was detected, either in the distal proliferative region or in meiotic prophase. However, H3K9me3 was still strongly present in germ cell nuclei throughout the gonad (Figure 5A and 5B and Figure S1). Therefore, MET-2 is required for adult germline H3K9me2 but not H3K9me3.

Similar results were obtained regardless of whether the worms examined were derived from a homozygous *met-2* strain or from a balanced heterozygous parent. The worms from the homozygous *met-2* mutant strain had neither a maternal contribution nor zygotic contribution of MET-2 (M−Z-), while *met-2* mutant worms obtained from a heterozygote parent potentially had maternally-derived MET-2 (M+Z-). As we could not detect H3K9me2 in either *met-2* M−Z- or M+Z- worms, we can conclude that maternal MET-2 does not contribute significantly to H3K9me2 in the adult germ line and that the loss of MET-2 for only one generation is sufficient to result in the absence of H3K9me2.

We also examined H3K9me2 in the germ lines of *met-2(n4256)* and *met-2(ok2307)* mutant males. We similarly found H3K9me2 to be undetectable, including on the single X chromosome (Figure 2C and Figure S1). Further, in *met-2(n4256)*; *him-8* double mutant hermaphrodites, both the enrichment of H3K9me2 on the unpaired X chromosomes and autosomal H3K9me2 were absent (Figure 2D). In spite of the loss of germline H3K9me2, germline H3K9me3 appeared normal. These data indicate that both endogenous germline H3K9me2, as well as H3K9me2 accumulation induced by defective pairing in the *him-8* mutant, is dependent upon MET-2. However, H3K9me3 in adult germ cells does not require MET-2.

**Figure 6:**
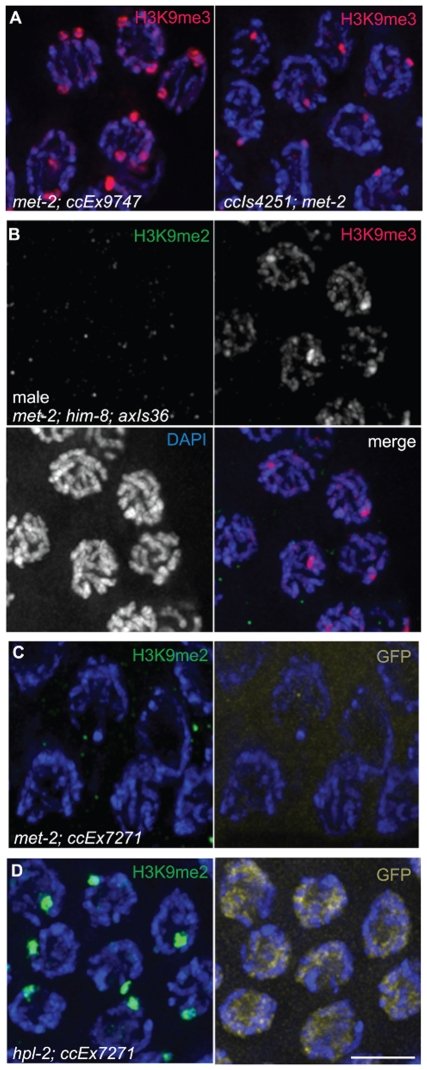
Effects of loss of MET-2 on high-copy arrays. (A) *met-2(n4256)*; *ccEx9747* and *ccIs4251*; *met-2(n4256)* hermaphrodite pachytene nuclei stained for H3K9me3. H3K9me3 is unaffected in *met-2* mutant worms. (B) Male *met-2(n4256)*; *him-8*; *axIs36* pachytene nuclei co-stained for H3K9me2 and H3K9me3. Despite the absence of H3K9me2, H3K9me3 is still restricted to the *axIs36* array on the X chromosome. (C) *met-2(n4256)*; *ccEx7271* pachytene nuclei stained with H3K9me2 and examined for *let-858::gfp* expression. Even without MET-2 and H3K9me2, the *let-858::gfp* transgene is still silenced in the germ line, demonstrated here by the lack of GFP visible in germline nuclei. (D) Control *hpl-2*; *ccEx7271* pachytene nuclei stained with H3K9me2 and examined for *let-858::gfp* expression. In the absence of *hpl-2*, H3K9me2 is still present on the array containing the *let-858::gfp* transgene, but *let-858::gfp* expression is desilenced in the germ line, as GFP is visible in the nuclei of germ cells. Red = H3K9me3, Green = H3K9me2, Yellow = GFP, Blue = DAPI. Scale bar = 5 µm.

The loss of *met-2* function also had no effect on H3K9me3 concentrated on high-copy transgene arrays. Both extrachromosomal (*ccEx9747*) and integrated (*ccIs4251*) arrays retained their enrichment for H3K9me3 in a *met-2(n4256)* mutant background (Figure 6A). Further, when we examined *met-2(n4256)*; *him-8* males carrying *axIs36* integrated into the X chromosome, we found that H3K9me3 was still present on *axIs36*, while the flanking endogenous X chromosome regions lacked H3K9me2. Despite the lack of H3K9me2, the array H3K9me3 remained restricted to a DAPI-faint region of the X chromosome and did not spread throughout the chromosome (Figure 6B).

We also tested whether the enrichment of H3K9me2 on extrachromosomal array *ccEx7271*, which contains the germline-competent (but silenced) *let-858::gfp* construct, was dependent on MET-2. As we found no detectable H3K9me2 in the germ lines of *met-2(n4256)*; *ccEx7271* worms (Figure 6C), the concentration of H3K9me2 on *ccEx7271* is MET-2 dependent. However, despite the loss of H3K9me2 enrichment on *ccEx7271*, we did not observe *let-858::gfp* expression in the germ lines of *met-2*; *ccEx7271* worms (Figure 6C), even though nuclear GFP was readily visible in somatic cells and embryos. GFP was still not apparent in *met-2*; *ccEx7271* germ line nuclei even after these worms were maintained as a homozygous strain for seven generations. Additionally, we easily detected *let-858::gfp* expression in the germ lines of *hpl-2*; *ccEx7271* worms, which lack a *C. elegans* homolog of heterochromatin protein HP1 that is required for germline silencing (Figure 6D and [31]). Therefore, we conclude that in *met-2*; *ccEx7271* mutant animals, *let-858::*GFP is not derepressed, and that the presence of MET-2 and/or H3K9me2 is not required to maintain germline transgene silencing. These data confirm and extend the previous observations that the reduction of H3K9me2 in both *ego-1* [32] and *him-17* [30] mutants fails to promote the derepression of *ccEx7271*.

### Gonadal Defects in *met-2* Mutant Worms

**Figure 7:**
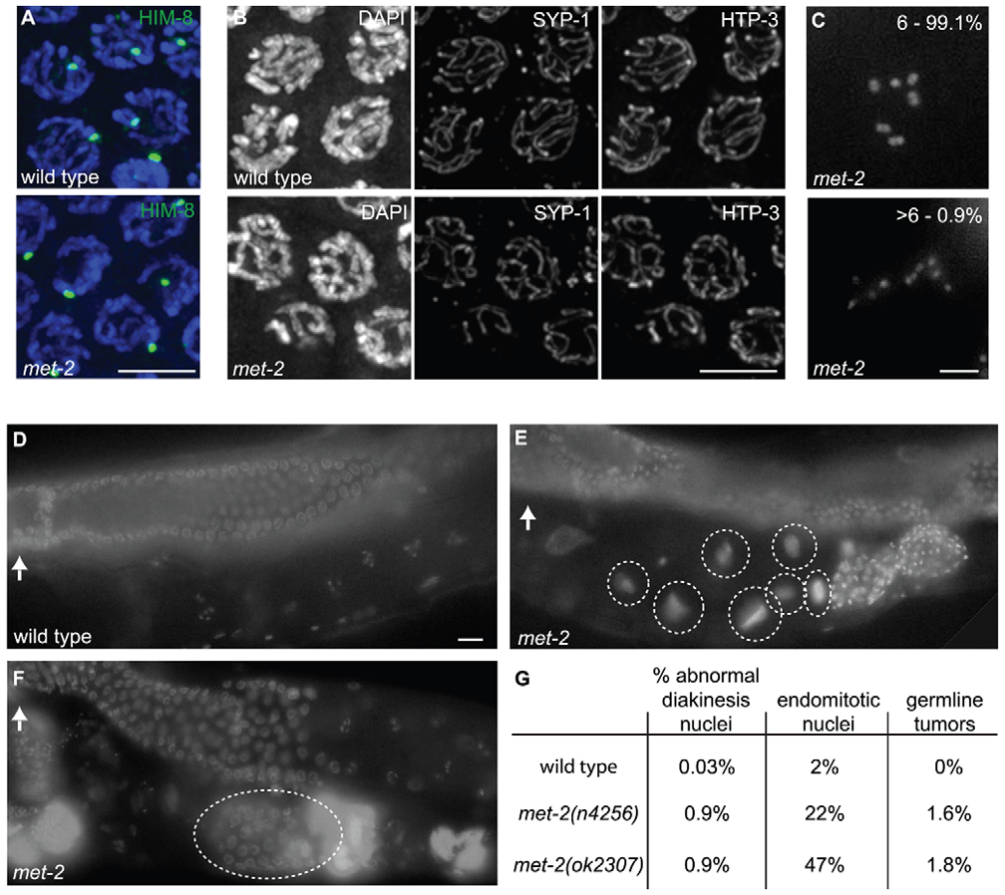
*met-2* phenotypes in the hermaphrodite gonad. (A) Wild type and *met-2* nuclei stained with HIM-8 antibody (Green). A single HIM-8 signal in each nucleus indicates that the X chromosomes in *met-2* mutant worms are paired. Blue = DAPI. Scale bar = 5 µm. (B) Wild-type and *met-2* pachytene nuclei co-stained for SC components SYP-1 and HTP-3. Chromosomes in *met-2* mutant worms appear to pair and synapse normally. Scale bar = 5 µm. (C) Diakinesis nuclei from *met-2* mutants. The top panel represents the 99.1% of *met-2* diakinesis nuclei with six DAPI-stained bodies, indicative of six pairs of homologous chromosomes held together by chiasmata. The bottom panel represents the 0.9% of *met-2* mutant diakinesis nuclei with greater than six DAPI-staining bodies, indicating that one or more chromosome pairs are not linked by chiasmata. Scale bar = 5 µm. (D) Ethanol fixed wild type whole worm, showing normal organization and progression of nuclei, from the distal germ line (marked with white arrow) to the proximal end of the germ line. Scale bar = 10 µm. (E) Ethanol fixed *met-2* whole worm with abnormal endomitotic nuclei (marked by white ovals). (F) Ethanol fixed *met-2* whole worm with a germ line tumor (indicated by the white oval). (G) Table summarizing the quantitation of *met-2(n4256)* and *met-2(ok2307)* hermaphrodite gonad phenotypes. All images are of *met-2(n4256)* animals.

Although a prominent histone modification is lost in adult germ cell nuclei of *met-2* mutant worms, many features of germline organization appeared largely normal in the vast majority of gonads examined. The DAPI-staining of germ lines from most *met-2* mutant worms revealed nuclei progressing normally from premeiotic proliferation to the diplotene stage of meiotic prophase I (Figure 5A). Additionally, experiments using the HIM-8 protein as a marker for successful X chromosome pairing indicated apparently normal homolog pairing in *met-2(n4256)* mutant worms (Figure 7A). Further, both chromosome synapsis, as assayed by immunolocalization of synaptonemal complex components SYP-1 and HTP-3 (Figure 7B), and progression of meiotic recombination as assayed by immunolocalization of the DNA strand exchange protein RAD-51 (data not shown) appeared normal in *met-2(n4256)* mutant worms. Finally, we found that most *met-2* nuclei in the diakinesis stage of meiotic prophase also appeared normal, containing six DAPI-stained bodies corresponding to the six pairs of *C. elegans* chromosomes attached by chiasmata, which are cytological indicators of successful crossover recombination.

While the above analysis indicated a high degree of success in progression through the meiotic program, we did detect evidence of meiotic prophase defects in a significant fraction of diakinesis-stage oocytes in *met-2* mutant hermaphrodites. Specifically, we found that 0.9% of *met-2(n4256)* diakinesis nuclei (n = 2275) and 0.9% of *met-2(ok2307)* diakinesis nuclei (n = 1135) contained greater than six DAPI stained bodies, whereas such nuclei were almost never observed in wild-type control hermaphrodites (1 of 2591 diakinesis nuclei; p<0.0001) (Figure 7C and 7G). The *met-2(n4256)* nuclei examined for the above experiment were obtained from a homozygous mutant strain over five generations (Table 2), and we did not detect any generational differences in the number of abnormal diakinesis nuclei present. The finding that *met-2* mutants exhibit an elevated but low frequency of meiotic defects indicates that while MET-2 is not strictly required for meiosis, it is required to ensure that the execution of the meiotic program will occur with high fidelity.

**Table 2:** Karyotype analysis of diakinesis-stage oocytes.

# of generations homozygous for *met-2(n4256)^b^*	Number of diakinesis nuclei with the indicated number of DAPI-stained bodies^a^						
	6	7	8	9	10	11	12
1 generation	1,043	5	0	0	0	0	0
2 generations	896	5	0	0	0	1	0
3 generations	189	6	1	0	0	0	0
4 generations	53	1	0	0	0	0	0
5 generations	94	1	0	0	0	0	0

a Worms were fixed and stained with DAPI as in [54]. The presence of six DAPI-stained bodies reflects successful chiasma formation for all homologs. The presence of more than six bodies indicates that some of the homologs have failed to properly form or maintain chiasmata.

b The distribution of DAPI-stained bodies does not show a trend from generation to generation.

In addition to the elevated occurrence of meiotic prophase defects, several additional phenotypes were detected in the reproductive tracts of *met-2* mutant worms. For example, 22% of *met-2(n4256)* adult hermaphrodites (n = 446) examined at 48 hours after the fourth larval stage contained endomitotic oocytes in the uterus, compared to 2% of wild type control worms (n = 438; p<0.0001) at the same stage. A high incidence of endomitotic oocytes was also seen in *met-2(ok2307)* worms (47% ; n = 220) (Figure 7D, Figure 1E and 1G). The increase in endomitotic oocytes seen in *met-2* mutant worms could indicate defective fertilization and/or premature depletion of sperm [33]. Further, germ line mitotic tumors were detected in 7 of 446 *met-2(n4256)* mutant worms and in 4 of 220 *met-2(ok2307)* mutant worms, but were not detected in wild type controls (0 of 438; p = 0.003 and 0.012) (Figure 7D, Figure 1F and 1G). The presence of germline tumors is consistent with elevated or ectopic proliferation signals and/or a heightened response to such signals [34]. These data contribute to the body of evidence that loss of *met-2* has pleiotropic consequences for multiple processes and tissues in *C. elegans* [29],[35],[36].

We also assessed cytological phenotypes of *met-2(n4256)*; *him-8* hermaphrodite germ lines. Most diakinesis nuclei in *him-8* single mutant control hermaphrodites contained 7 DAPI-stained bodies, reflecting lack of crossing over between the unpaired X chromosomes but successful pairing and recombination for the autosomes; only 1% of nuclei had >7 DAPI bodies (n = 600). The frequency of diakinesis nuclei with >7 DAPI bodies was increased to 3% in *met-2*; *him-8* hermaphrodites (n = 635; p = 0.015). Additionally, 4% of *met-2*; *him-8* animals (n = 143) examined had germline tumors, while tumors were not seen in *him-8* controls (0 of 131; p = 0.03).

### Loss of MET-2 and H3K9me2 Does Not Reduce the Efficiency of Transmission of X Chromosomes during Spermatocyte Meiosis

The high concentration of H3K9me2 on the single X chromosome during male meiosis raised the possibility that the H3K9me2 chromatin modification might be important for the faithful transmission of this naturally partnerless chromosome during the spermatocyte meiotic divisions. If male X chromosome transmission were perturbed, it would result in an increased frequency of nullo-X sperm (*i.e.*, sperm that lack an X chromosome). Therefore, we tested this possibility by assessing the sex ratios among the progeny of *met-2* mutant and control males. Specifically, we set up single male matings using marked hermaphrodites to enable identification of cross progeny. The fraction of male cross progeny produced did not differ significantly between the *met-2* mutant crosses (51.7% males, n = 2178 total cross progeny) and either *+/+* (50.7% males, n = 2070) or *+/met-2* (50.6% males, n = 1307) control crosses. Therefore, loss of MET-2 and consequent loss of H3K9me2 does not significantly impair the efficiency of transmission of the single X chromosome during male spermatocyte meiosis.

As H3K9me2 is heavily enriched on the unpaired X chromosomes in *him-8* hermaphrodites, we also conducted an experiment aimed at testing whether the efficiency of X chromosome transmission might be impaired during spermatocyte meiosis in *met-2*; *him-8* hermaphrodites. Previous analysis of *him-8* mutant hermaphrodites had shown that crossing over between the X chromosomes is impaired during both oocyte and spermatocyte meiosis, but a high frequency of X chromosome loss and mis-segregation occurs only during oocyte meiosis [37],[38]. Based on these and other observations, it has been inferred that *C. elegans* hermaphrodites may have a capacity to segregate achiasmate X chromosomes during spermatocyte meiosis [37]–[39]. Thus, we assessed whether loss of *met-2* function might impair the ability to transmit achiasmate X chromosomes during hermaphrodite spermatocyte meiosis. Specifically we asked whether *met-2*; *him-8* double mutant hermaphrodites had an increased frequency of male self-progeny compared with *him-8* single mutant hermaphrodites, as might be expected if X chromosomes were now being lost during spermatocyte meiosis as they are during *him-8* oocyte meiosis. This analysis failed to find evidence for elevated X chromosome loss during spermatocyte meiosis, as the fraction of male self-progeny produced by *met-2*; *him-8* hermaphrodites (34.7%, n = 1734) was not increased over that observed for *him-8* controls (37.5%, n = 2085, p = 0.08).

### H3K9me3 Is Reduced in *mes-2* Mutant Germ Lines

**Figure 8:**
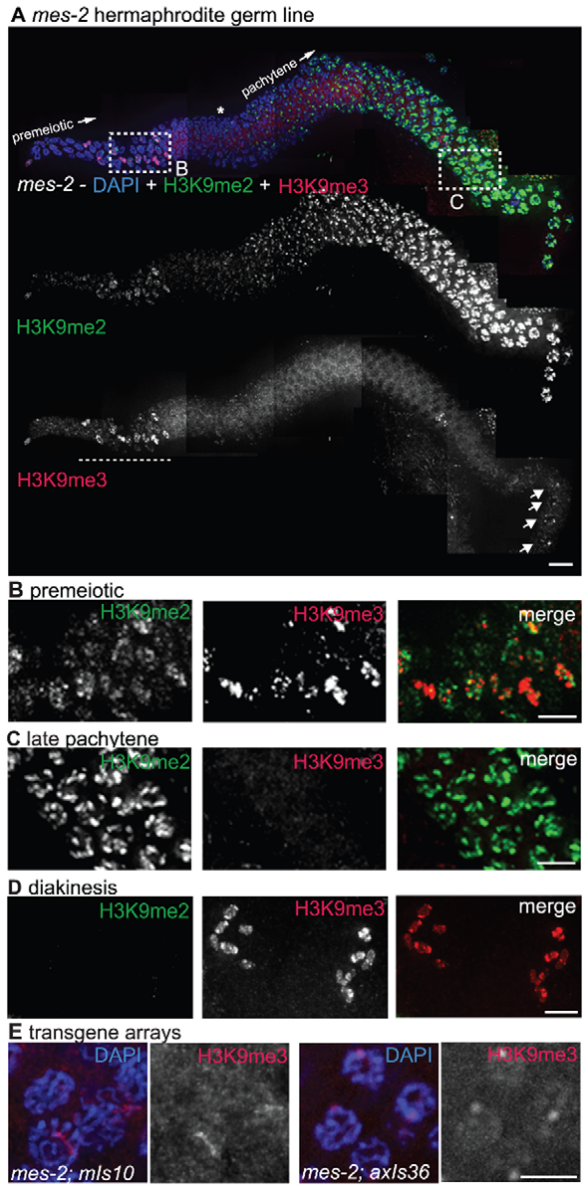
*mes-2* is required for wild-type H3K9me3 levels. (A) A germ line dissected from an M+Z- *mes-2 C. elegans* hermaphrodite. The premeiotic region of the germ line is on the left, while germ cells at the diplotene stage of meiotic prophase are on the right. Top: anti-H3K9me2 (green) and anti-H3K9me3 (red) staining overlaid on DAPI-stained chromosomes (blue). Boxed regions are enlarged in (B,C). Middle: anti-H3K9me2 staining alone. Bottom: anti-H3K9me3 staining alone. Most H3K9me3 staining is absent in the *mes-2* mutant, however DAPI-stained bodies thought to be mitotic figures still stain for H3K9me3 (underlined with dashed line) and diplotene nuclei retain some anti-H3K9me3 staining (arrows). An asterisk indicates the approximate position of meiotic prophase entry, while the presence of a single line of nuclei indicates the beginning of the diplotene stage. Scale bar = 10 µm. (B) Enlargement of the premeiotic region from A, showing that H3K9me3 is still present on chromatin that appears to correspond to mitotic figures. Scale bar = 5 µm. (C) Enlarged pachytene nuclei from A, showing that while H3K9me2 is unaffected, most nuclei lack H3K9me3 staining. Scale bar = 5 µm. (D) Representative *mes-2* diakinesis nuclei. Even though H3K9me3 staining is reduced or lost from the majority of germ cells, diakinesis nuclei in the *mes-2* mutant usually retain H3K9me3 staining. The absence of H3K9me2 from diakinesis-stage chromosomes seen here is a normal feature of germ lines. Scale bar = 5 µm. (E) *mes-2*; *mIs10* and *mes-2*; *axIs36* stained with anti-H3K9me3 and DAPI. While most endogenous H3K9me3 staining is lost, the transgene arrays can retain reduced H3K9me3. Scale bar = 5 µm.

As MET-2 is not required for H3K9me3 accumulation in the adult germ line, we investigated the possibility that a different germline histone methyltransferase (HMTase), MES-2, might play a role in the acquisition or maintenance of this mark. MES-2 is required for other repressive modifications, namely H3K27me2 and H3K27me3, in proliferating germ cells and pachytene nuclei in the adult *C. elegans* germ line [6]. *mes-2* mutant worms derived from a heterozygous mother (M+Z-) appear largely normal and are fertile, but their offspring (M−Z-) are sterile and develop with an abnormal and degraded germ line [18]. As previously reported [6], we found that H3K9me2 appeared normal in *mes-2* M+Z- germ lines. In contrast, we observed that much of the germline signal detected by the H3K9me3 antibody was dependent on MES-2. Specifically, we found that 74% (25 of 34) of germ lines from M+Z- hermaphrodites carrying the *mes-2(bn11)* allele showed reduced or absent H3K9me3 staining in most nuclei from the distal proliferative region through late pachytene (Figure 8; Figure S2). However, in all *mes-2(bn11)* mutant hermaphrodites examined, we consistently detected strong residual H3K9me3 staining remaining on chromosomes in diplotene and diakinesis stage germ cells, as also occurs for the H3K27me2/3 modifications (Figure 8A and 8D and [6]). Additionally, H3K9me3 was still present on condensed chromatin in the mitotic region, which likely corresponds to mitotic figures (Figure 8A and 8B). These data indicate that a subset of germline H3K9me3 staining is MES-2-independent.

The incomplete penetrance and variability in germ line H3K9me3 reduction observed for the *mes-2(bn11)* mutant appears to be an inherent property of mutants lacking MES-2 function, as similar results were obtained for worms carrying the *mes-2(bn27)* allele (8 of 15 germ lines examined showed a loss or reduction in H3K9me3) (Figure S2). We also obtained similar results when we examined H3K9me3 staining in worms mutant for *mes-6*, which encodes a component of the MES-2/MES-3/MES-6 protein complex that was previously shown to be required *in vivo* for production of the MES-2-dependent H3K27me2/3 marks [6]. 75% (15/20) of the *mes-6* (M+Z-) gonads examined showed an absence or reduction of H3K9me3 in most germ cells (Figure S2). The reduction of H3K9me3 staining is not, however, a general property of *mes* mutant strains, as loss of H3K9me3 was not detected in either *mes-4(bn67)* or *mes-4(bn23)* mutant germ lines (Figure S2). These findings are consistent with the possibility that the same protein complex responsible for H3K27 trimethylation may also promote, either directly or indirectly, normal acquisition or maintenance of H3K9me3.

We also examined H3K9me3 staining in *mes-2(bn11)* M+Z- mutant worms carrying either the *mIs10* (V) or the *axIs36* (X) high-copy transgene array. In the majority of germ lines examined, H3K9me3 was lost from most of the endogenous chromosomes, and array-associated H3K9me3 staining was reduced compared to control germ lines (Figure 8E versus Figure 3A and 3D).

Although array-associated H3K9me3 staining was usually reduced, some H3K9me3 enrichment on the arrays was often still detected. This result suggests either that array H3K9me3 is not entirely dependent on MES-2 or that even without new H3K9me3 being generated, H3K9me3 on high-copy arrays cannot be reduced to undetectable levels in only one generation.

### Lack of Synthetic Phenotypes in *met-2*; *mes-2* Double Mutant Germ Lines

We have demonstrated that H3K9me2 and H3K9me3 exhibit distinct localization patterns and are acquired independently in the adult germ line. Thus, although both of these modifications are considered to be hallmarks of transcriptional repression [40],[41], our data are consistent with the hypothesis that they function independently. To investigate this possibility, we examined the phenotype of *met-2*; *mes-2* double mutants.

For these experiments, we constructed a strain in which both mutations were maintained in a heterozygous state. We found that homozygous *met-2*; *mes-2* double mutant worms derived from this strain did not exhibit any synthetic phenotypes, in either the M+Z- or M−Z- generations. Like *mes-2* single mutants, M+Z- *met-2*; *mes-2* double mutant worms appeared normal and were fertile but produced sterile M−Z- offspring with degraded germ lines. This was true whether the two mutations became homozygous at the same generation or whether the *met-2* mutation had been maintained in a homozygous state for up to 6 generations before the *mes-2* mutation became homozygous. The fact that we did not detect any synthetic phenotypes in *met-2*; *mes-2* double mutant worms indicates that adult germline H3K9me2 is unlikely to be functionally redundant with H3K9me3 and/or H3K27me2/3, consistent with the differential localization exhibited by these modifications.

## Discussion

### H3K9me2 and H3K9me3 Are Largely Independent Modifications

In this work we have examined the localization and acquisition of the H3K9me2 and H3K9me3 histone modifications in the adult *C. elegans* germ line. Although both of these marks are considered to be repressive modifications and occur at the same residue on histone H3, we have demonstrated that these marks are largely independent. First, we showed by co-immunofluorescence with an anti-H3K9me2 monoclonal antibody and an anti-H3K9me3 polyclonal antibody that these modifications exhibit divergent germline localization patterns. Second, we demonstrated that MET-2 is required for the presence of H3K9me2 in the germ line but is dispensable for H3K9me3. Conversely, MES-2 is required for much of the H3K9me3 but is dispensable for germline H3K9me2. Finally, consistent with the independent localization and acquisition of the two marks, there is a lack of synthetic defects in *mes-2*; *met-2* double mutant worms.

As our work is the first thorough examination of H3K9me3 localization in the *C. elegans* germ line, this study provided the first opportunity to compare the germline distributions of H3K9me3 and H3K9me2. We have demonstrated here that H3K9me3 is present on all chromosomes in all germ cells in *C. elegans* hermaphrodites and can be visualized until the primary spermatocyte stage in males. This broad chromosomal distribution means that the H3K9me3 signal sometimes overlapped with the more restricted H3K9me2 signal. However, the peaks of H3K9me3 staining rarely overlapped with the brightest regions of the H3K9me2 staining pattern, emphasizing the largely divergent nature of these modifications. Additionally, the presence of some staining overlap indicates that the dissimilar patterns were not likely caused by one antibody excluding the other from chromatin.

An understanding of the functions of and relationships between the H3K9me2 and H3K9me3 chromatin modifications is being pursued through ongoing work in several different experimental systems. One of the hypotheses to emerge from these analyses is that H3K9me3 corresponds to the presence of constitutive heterochromatin, while H3K9me2 is enriched on silenced euchromatin [23],[40],[42],[43]. Consistent with the idea that H3K9me2 and H3K9me3 are associated with repression, in *C. elegans* previous cytological experiments have correlated the presence of H3K9me2 with chromatin thought to be subjected to silencing, including germline-silenced transgene arrays and the weakly transcribed single sex chromosome in males [9]. H3K9me2 has also been seen to concentrate on unpaired/unsynapsed chromosomes in certain meiotic mutants, but the transcriptional states of these chromosomes is not known [13]. More recently, the presence of H3K9me3 has been correlated with silenced chromatin in *C. elegans*, as ChIP/chip experiments using *C. elegans* larvae have shown H3K9me3 to be present at genes that are not expressed or are expressed at very low levels [12].

Our analysis of the localization patterns of H3K9me2 and H3K9me3 in the *C. elegans* adult germ line indicates that H3K9me2 and H3K9me3 are associated predominantly with different chromatin domains exhibiting distinct cytological and functional properties. These findings are compatible with the aforementioned *C. elegans* data and the presence of at least two independent transcriptional repression mechanisms. However, our data also suggest that at least some of the roles for H3K9me2 and H3K9me3 in the *C. elegans* germ line could be different than those proposed for other systems. For example, the broad chromosomal distribution of H3K9me3, together with the correspondence of H3K9me3 to DAPI-faint DNA, does not support the notion that H3K9me3 is restricted to cytologically-defined heterochromatin in the *C. elegans* adult germ line. Further, the enrichment of H3K9me2 on specialized chromatin structures (*i.e.* germline silenced extrachromosomal arrays and unpaired/unsynapsed sex chromosomes) suggests that H3K9me2 may have additional functions distinct from silencing of euchromatic genes; some of these additional functions may be conserved, as mammalian sex chromosomes also become enriched for H3K9me2 during spermatocyte meiosis [44].

Our finding that H3K9me2 is not detectable in the adult germ lines of *met-2* mutant worms supports the conclusion that MET-2 is likely the HMTase directly responsible for H3K9me2 in the *C. elegans* germ line. Although MET-2 histone methyltransferase activity could not be assessed directly due to the insolubility of the MET-2 protein (ECA and JBB, unpublished), several other members of the same HMTase family have demonstrated methyltransferase activities *in vitro* [25]–[28]. Our data also demonstrated that MET-2 is not required for H3K9me3 in the adult germ line, indicating that another HMTase must be responsible for H3K9me3 in adult germ cells. This result was unanticipated based on previous data showing that H3K9me3 levels, as measured by western blot analysis, are reduced by 70–80% in *met-2* mutant embryos [29]. However, the residual H3K9me3 detected in this prior analysis indicates that another HMTase must also be responsible for a significant fraction of H3K9me3 in embryos. It remains an open question whether the reported reduction of H3K9me3 in embryos is an indirect consequence of MET-2 loss or reflects different specificities for MET-2 in different tissues. There is precedent for a single HMTase being required for different marks in different tissues, as dSETDB1, which is a member of the same HMTase family as MET-2, has been shown to be required for H3K9me2 accumulation on chromosome 4 in *Drosophila* salivary glands [26],[45], while it is required for H3K9me3, but not H3K9me2, in germline stem cells and their immediate descendants [46].

Even though germ line H3K9me2 and H3K9me3 are often differentially localized, several methylation models propose that the trimethyl mark is built upon a dimethyl mark [27],[47]. However, our results do not support this hypothesis, because when H3K9me2 is absent in *met-2* mutant germ lines, the appearance of the H3K9me3 modification is indistinguishable from wild type germ lines. While it is possible that a transient dimethyl mark is required for the formation of H3K9me3, the differential localization of these modifications indicates that any transient H3K9me2 is likely to be independent of the H3K9me2 visualized in the germ line.

While acquisition of H3K9me2 in adult germ cells requires MET-2, we found that H3K9me3 is partially dependent upon MES-2, a HMTase previously shown to be required for H3K27me2 and H3K27me3 in the *C. elegans* germ line. MES-2 is related to E(z), a HMTase characterized as being required for the methylation of H3K27 in *Drosophila* [48]. While the dependence of H3K9me3 on MES-2 could be due to an indirect mechanism, early biochemistry done with the E(z) protein showed the potential for proteins in this family to have both H3K9 and H3K27 methylase activity [47],[49]. This work suggests that the MES-2 HMTase activity could be directly responsible for H3K9me3. Currently, our data cannot differentiate between direct and indirect mechanisms for MES-2 in K9 methylation.

While we saw a clear reduction in the intensity of H3K9me3 staining in most *mes-2* mutant germ lines examined, H3K9me3 was not completely absent. This observation is consistent either with an inefficient, indirect role for MES-2 in the production of H3K9me3 or with the presence of a second H3K9me3 methyltransferase activity in the adult germ line, as has been proposed for H3K27me2/3 [6]. Alternatively, even though there is a known demethylase that can remove H3K9me3 from *C. elegans* chromosomes [19], this activity may be inefficient, consequently allowing H3K9me3 to persist even after the HMTase responsible for the mark has been lost. Interestingly, a partial chromatin defect resulting from the absence of *mes-2* has been previously observed, as loss of *mes-2* results in a reduction but not complete loss of complex transgene array compaction in the *C. elegans* embryo [24].

### Properties of High-Copy Transgene Arrays in *C. elegans*

One of the most striking differences in the distribution of H3K9me2 and H3K9me3 was revealed through an analysis of high-copy transgene arrays. High-copy transgene arrays are a widely used tool for studying gene transcription patterns and for rescuing mutant phenotypes of genes that function in the *C. elegans* soma. In this work, we found that high-copy arrays carrying transgenes with several different somatic-cell specific promoters were highly enriched for H3K9me3, and this enrichment was observed both for extrachromosomal arrays and arrays integrated at multiple positions in the genome.

A possible function for the H3K9me3 modification on arrays could be to solidify the identity of the array as being different from the native DNA and therefore prevent the array from interfering with the functions of the rest of the genome. As integrated high-copy arrays are derived from extrachromosomal DNA elements, and extrachromosomal arrays are also enriched for H3K9me3, this modification is likely acquired before genomic integration occurs. Further, arrays are initially assembled from hundreds of separate DNA fragments, including bacterial plasmids, which are introduced by microinjection into the germ line of the founding parent animal. Therefore, the enrichment of H3K9me3 could be in response to the large amount of foreign DNA present in these arrays, to the creation of unusual array DNA/chromatin structures and/or to the presence of genes with promoters not normally expressed in the germ line.

Alternatively, or in addition, the presence of enriched H3K9me3 could indicate that high-copy arrays composed of soma-specific promoters are subjected to silencing. While images shown here depict H3K9me3 enriched on high-copy arrays in the germ line, we also detected domains of H3K9me3 enrichment in the nuclei of somatic cells in worms carrying these high-copy arrays (Figure S3). This enrichment in somatic cells could provide insight into the perplexing issue of why high-copy transgene arrays work as well as they do for rescue and expression experiments despite carrying hundreds of copies of their component transgenes. H3K9me3 is likely repressive, as this modification was recently shown by ChIP/chip analysis to be enriched on low-expressed or non-expressed genes in *C. elegans* larvae [12]. Therefore, the high-levels of H3K9me3 on high-copy transgene arrays may mean that most copies of the transgenes are not expressed or are expressed at very low levels, and/or that only a few transgene copies actually contribute to the observed expression.

For several organisms, the presence of H3K9me3 is reported to indicate the presence of heterochromatin, often defined by the presence of dense, DAPI-bright chromatin. However, in the *C. elegans* germ line, while the enrichment of H3K9me3 on high-copy arrays might indicate that silencing is occurring, the corresponding chromatin has an open, DAPI-faint appearance. This DAPI-faint appearance could be indicative of the array adopting a novel chromatin/DNA structure or of *C. elegans* heterochromatin adopting a configuration distinct from that seen in other organisms.

The DAPI-faint appearance of high-copy arrays was observed both when arrays were present on paired and synapsed chromosomes as well as when arrays were present on unpaired X chromosomes. Unpaired X chromosomes have been shown to become heavily enriched for H3K9me2, and this enrichment is hypothesized to be part of a mechanism that can sense and respond to the presence of unpaired chromosomes in meiosis [13]. However, we found that the high-copy arrays present on the unpaired X chromosomes in *him-8*; *axIs36* hermaphrodites retained an enrichment of H3K9me3 and failed to become dimethylated. This observation could result from an unusual DNA/chromatin structure and/or the presence of H3K9me3 preventing the unpaired arrays from acquiring H3K9me2. This latter possibility could be due to either H3K9me3 blocking the formation of H3K9me2 or because chromatin enriched for H3K9me3 is not recognized as being unpaired. While H3K9me3 could render the accumulation of H3K9me2 unnecessary, these results indicate that the presence of unpaired chromosome segments alone is not sufficient to initiate the dimethylation of chromatin during *C. elegans* meiosis.

The only high-copy array in our analysis that was not enriched for H3K9me3 was an extrachromosomal array composed of multiple copies of a *let-858::gfp* transgene, which contains a germline-competent promoter that is silenced in the germ line. Instead this array is enriched for H3K9me2, and it has been proposed that H3K9me2 plays a role in the silencing of *let-858::gfp* [9]. However, the fact that *let-858::gfp* silencing is maintained in *him-17* and *ego-1* mutants where the acquisition of H3K9me2 is reduced and/or delayed has suggested either that H3K9me2 is not required for silencing or that reduced levels of H3K9me2 were sufficient for silencing [30],[32]. Here, we show that the loss of the HMTase responsible for H3K9me2 also does not result in the loss of *let-858::gfp* silencing, demonstrating that neither MET-2 nor H3K9me2 is required for the maintenance of transgene silencing. However, our data do not rule out the possibility that H3K9me2 could still be required for the establishment of silencing.

### The Role of H3K9me2 in the Adult Germ Line

Despite the prominent and highly dynamic localization of the H3K9me2 mark in the *C. elegans* germ cells, loss of MET-2 and consequently the loss of H3K9me2 had remarkably little visible impact on germ line organization and the chromosomal events of meiosis in most germ lines examined. Further, loss of the prominent H3K9me2 accumulation on unpaired sex chromosomes in males and *him-8* hermaphrodites did not result in elevated X chromosome loss during spermatocyte meiosis. However, the 1% occurrence of achiasmate chromosomes at diakinesis demonstrates that MET-2 could function to ensure the fidelity of chromosome segregation during meiosis. That is, although MET-2 activity is not strictly required to execute any of the events of meiotic prophase, MET-2 is required to ensure that these events occur with high fidelity.

There are several ways in which MET-2 and/or H3K9me2 could function to maintain meiotic fidelity. In worms lacking these factors, chromosome and chromatin structures could be altered, resulting in an error-prone meiosis due to differences in meiotic protein accessibility or an alteration in the ability to repair meiotic recombination intermediates. Another possibility is that without MET-2 and/or H3K9me2, nuclei that should have been culled from the germ line due to infrequent meiotic errors might escape detection and/or elimination by meiotic checkpoints and go on to form abnormal diakinesis nuclei [50],[51]. Alternatively, loss of MET-2/H3K9me2 might affect the transcription levels of some genes required for normal entry into, progression through and/or execution of the meiotic program. Under this scenario, the low penetrance of the observed meiotic defects could be caused by a relatively modest perturbation of gene expression levels and/or by the buffering effects of other regulatory mechanisms that operate in parallel to ensure a robust outcome for meiosis.

MET-2 has been previously implicated as a factor required for transcriptional repression in vulval development. However, the role of MET-2 in this process was not evident from a phenotypic analysis of *met-2* single mutants alone, but was revealed when *met-2* function was eliminated in combination with another protein involved in vulval cell differentiation. This property identifies *met-2* as a synthetic multivulva (synMuv) gene [29],[52]. By definition, the synMuv phenotype can only occur when the activities of two genes are reduced or eliminated simultaneously; therefore the synMuv phenomenon has been interpreted to reflect the presence of at least two parallel pathways that collaborate to ensure the accurate specification and differentiation of vulval precursor cells (reviewed in [53]). The fact that many identified synMuv proteins are associated with chromatin and/or are components of conserved chromatin remodeling or modifying complexes suggests that such complexes are individually dispensable for executing distinct cellular programs, but instead function redundantly to ensure reliable outcomes. By analogy, the low penetrance of defects observed when MET-2 is absent from the adult germ line may not necessarily be an accurate reflection of the importance of MET-2 function in adult germ cells, and future analysis may reveal factors that function in parallel with MET-2 to ensure normal progression through the meiotic program and successful execution of the chromosomal events required for faithful sexual reproduction.

## Materials and Methods

### Genetics

Except where specified, all *C. elegans* strains were cultured at 20°C under standard conditions. The following mutations, balancers and transgenes were used:

*Chromosome I*: *dpy-4(hc40)*, *ccIs4251*[*myo-3::Ngfp-lacZ myo-3::Mtgfp*], *ccIs9753* [*myo-2::GFP*; *pes-10::gfp*; *F22B7.9::gfp*]

*Chromosome II*: *mes-2(bn11)*, *mes-2(bn27)*, *unc-4(e120)*, *mnC1*[*dpy-10(e128) unc-52(e444)*] *Chromosome III: qC1*[*dpy-19(e1259) glp-1(q339) qIs26(lag-2::GFP)*], *met-2(n4256)*, *met-2(ok2307)*, *hpl-2(tm611)*

*Chromosome IV*: *zim-2(tm574)*, *him-8(tm611)*, *mes-6(bn66)*, *dpy-20(e1282ts)*, *spe-8(e1166)*, *nT1*[*unc?(n754) let-?*] IV:V

*Chromosome V*: *dpy-11(e224)*, *mes-4(bn23)*, *mes-4(bn67)*, *unc-76(e911)*, *mIs10*[*myo-2::gfp*; *pes-10::gfp*; *F22B7.9::gfp*], *nT1* IV:V

*Chromosome X*: *axIs36*[*pes-10::gfp*]

*extrachromosomal elements*: *ccEx7271*[*let-858::gfp, pha-1(+)*], *ccEx9747*[*myo-2::gfp*; *pes-10::gfp*; *F22B7.9::gfp*]

The *met-2(n4256)* deletion strain was described in [29]. The *met-2(ok2307)* deletion was generated by the *C. elegans* Gene Knockout Consortium and contains a deletion of 1320 bp, from coordinates 8378356 to 8379675 on chromosome III that removes part of the SET domain, the protein region linked to methyltranferase activity. Most analysis was performed using the *met-2(n4256)* mutation, which was maintained as a balanced heterozygote *met-2/qC1*; *met-2(n4256)* had been outcrossed previously by ECA and was outcrossed at least four more times by JBB). *met-2(ok2307)* was outcrossed two times and was also maintained as *met-2/qC1*. Except where otherwise stated, analysis using *met-2* mutant worms was done with *met-2(n4256)* worms of the M+Z- generation. All *met-2* images in Figure 1, Figure 2, Figure 3, Figure 4, Figure 5, Figure 6, Figure 7, Figure 8 are of *met-2(n4256)* animals.

*mes-2* mutations were linked to *unc-4(e120)* and maintained in a strain with *mnC1*. *mes-6(bn66)* was linked to *dpy-20(e1282ts)* and maintained in a strain with *nT1*. *mes-4(bn23)* was linked to both *dpy-11(e224)* and *unc-76(e911)* and maintained in a strain with *nT1*, while *mes-4(bn67)* was linked to *dpy-11(e224)* and was also maintained in a strain with *nT1*. Analysis of *mes* mutant worms was done using the M+Z- generation, unless otherwise stated.

For quantitation of *met-2* diakinesis defects, germline tumors, and endomitotic oocytes, M+Z- L4 worms were picked to individual plates and transferred to new plates at 24 hours. At 48 hours post L4, the worms were fixed with ethanol and stained with DAPI to examine meiotic nuclei [54]. M−Z- L4s were picked to their own plates and the analysis was repeated; this process was repeated for several subsequent generations as indicated in the Results section.

For the analysis of X chromosome transmission *met-2(n4256)* or control males were mated to *spe-8(e1166)*; *dpy-4(hc40)* hermaphrodites; cross progeny were identified as non-Dpy males and hermaphrodites. We had difficulty generating and maintaining homozygous *met-2* mating strains, suggesting that M−Z- *met-2* males do not mate efficiently. Thus we generated M+Z- *met-2* males for use in these experiments. These males were generated using series of crosses that produced *met-2/met-2* and *met-2/+* males in equal proportions. Individual male matings were conducted, and males were genotyped by PCR after they had mated.

### Cytological Analysis

Except where noted below, fixation, DAPI-staining, immunostaining and acquisition and processing of images using the Deltavision deconvolution microscopy system was carried out as described in [30], using gonads dissected from 20 hour post-L4 adults (see Text S1). The following primary antibodies were used at the indicated dilutions: H3K9me2 mouse monoclonal from Abcam – ab1220 lot #471913 (1∶500); H3K9me3 rabbit polyclonal from Abcam – ab8898 lot #339900 (1∶500); H3K27me3 rabbit polyclonal from Upstate – 07-449 lot #DAM1387952 (1∶200); chicken HTP-3 (1∶500) [55]; guinea pig HIM-8 (1∶500) [56]; guinea pig SYP-1 (1∶200) [57]. Secondary antibodies used were Alexa 488 α-mouse, Alexa 488 α-rabbit, Alexa 555 α-rabbit, Alexa 555 α-chicken, Alexa 488 α-guinea pig (all 1∶500). For experiments where double labeling was done, the primary antibodies were applied simultaneously, followed by a simultaneous application of secondary antibodies. Images were collected in Z-series representing a longitudinal bisection of each gonad arm; images shown are projections through data stacks encompassing whole nuclei.

For the H3K9me2 experiments, we and others had found that previously purchased polyclonal antibodies were inconsistent from experiment to experiment. In contrast, we found that the mouse monoclonal antibody ab1220 gave highly reproducible results by IF. We also tested ab1220 in western analysis. Using a mixed-stage whole worm protein preparation and a chromatin fraction generated from whole adult *C. elegans* [58], ab1220 detected a single band of the appropriate size to be a histone; further, ab1220 produced no signal when applied to a western blot of a non-chromatin fraction. Therefore, we concluded that ab1220 is a highly specific antibody. We used ab1220 to generate all anti-H3K9me2 images in this paper.

For the H3K9me3 experiments, several antibodies were tested. Antibodies that were a gift from David Allis were originally used for staining the transgene arrays but were in short supply, so we sought an alternative. Antibodies that were from a gift from Thomas Jenuwein duplicated our results but did not penetrate the *C. elegans* germ line well; similar results were obtained with Upstate 07-442, lot# JBC1361819. Abcam ab6001 failed to show any staining in *C. elegans*. Finally, we found that Abcam antibody ab8898 reproduced our results and consistently penetrated the germ line well, making it a highly reliable reagent. When Abcam ab8898 was applied to a western blot of whole animal, mixed-stage *C. elegans* protein extract, two bands were detected, the expected histone-size band and a second higher molecular weight band. However, only the histone-size band was detected on western blots of a chromatin fraction from an adult *C. elegans* extract. This result indicates that the ab8898 IF signal that colocalizes with DAPI-stained chromatin in the adult germ line is specific to histones. All anti-H3K9me3 images in this publication were generated using ab8898.

The procedure for combined immunofluorescence and fluorescence *in situ* hybridization was modified from ([9] and K. Nabeshima, pers. comm.). The FISH probe was made from 3 µg of a plasmid carrying a *sur-5::gfp* transgene. This plasmid was digested using numerous 4 base-pair cutting restriction enzymes. DNA was then precipitated, resuspended and labeled as per instructions from the *ULYSIS Nucleic Acid Labeling Kit* from Molecular Probes, using 2 µg of DNA per labeling reaction. After labeling, the reactions were cleaned using CentriSep Spin Columns from Princeton Separations. 2 µl of the volume recovered from the column was used to probe one FISH slide.

Images in Figure 7C–7F were generated by fixing whole adult worms 48 hours post-L4, using ethanol fixation [54]. Images were collected using a standard fluorescence microscope with a CCD camera.

Images in Figure S3 were generated using the Deltavision microscopy system, however the deconvolution algorithm was not applied. Intestine was dissected from adult worms and the embryos analyzed were both from eggs dissected from adult worms as well as from eggs laid onto plates.

## Supporting Information

Figure S1 *met-2(ok2307)* is required for H3K9me2 in the adult germ line. (A) A germ line dissected from a *met-2(ok2307) C. elegans* hermaphrodite. The premeiotic region of the germ line is on the left, while germ cells at the diplotene stage of meiotic prophase are on the right. Top: anti-H3K9me2 staining (green) and anti-H3K9me3 staining (red) overlaid on DAPI-stained chromosomes (blue). Middle: anti-H3K9me2 staining alone. Bottom: anti-H3K9me3 staining alone. No chromosomal H3K9me2 staining is visible in the *met-2(ok2307)* mutant germ line. (B) *met-2(ok2307)* male pachytene germ cell nuclei, stained with anti-H3K9me2 and anti-H3K9me3 antibodies. In the pachytene nuclei from the *met-2(ok2307)* mutant male, H3K9me2 is no longer detected on the unpaired male X chromosome, while the distribution of H3K9me3 is unaffected. Blue = DAPI, Green = H3K9me2, Red = H3K9me3. Scale bar = 10 µm.
(1.61 MB TIF)

Figure S2 Status of H3K9me3 in the germ cells of *mes* mutants. Wild type, *mes-2(bn11)*, *mes-2(bn27)*, *mes-4(bn23)*, *mes-4(bn67)* and *mes-6(bn66)* germ lines categorized with respect to H3K9me3 staining of germ cell nuclei from the premeiotic region through to the end of the pachytene stage. Panels at the top of the figure depict pachytene nuclei representing each of the three staining categories used. Germ line staining was categorized as “present” when the H3K9me3 staining was almost indistinguishable from wild type H3K9me3 staining. Germ line staining was classified as “reduced” in cases where the H3K9me3 signal was clearly fainter and readily distinguishable from that seen in wild-type germ lines, but individual nuclei could still be discerned based on anti-H3K9me3 staining. Germ line staining was classified as “absent” when chromosomal H3K9me3 was indistinguishable from background levels.
(0.78 MB TIF)

Figure S3 H3K9me3 marks *mIs10* in embryos and adult intestine. (A) A *mIs10*∼16 cell embryo. Bright regions of *mIs10*-dependent H3K9me3 staining are apparent in all embryonic cells. (B) A *met-2*; *mIs10*∼22 cell embryo. In the *met-2*; *mIs10 embryo*, *mIs10*-dependent H3K9me3 staining is still detected. (Nuclei at the far right of the images are not part of the embryo). (C) A polyploid intestinal nucleus from an adult *mIs10* hermaphrodite. Two regions enriched for H3K9me3 staining are present, corresponding to the presence of the *mIs10* array. (D) A polyploid intestinal nucleus from an adult *met-2*; *mIs10* hermaphrodite; two *mIs10*-dependent H3K9me3 signals are still detected. Embryos and intestinal nuclei have been co-stained with anti-H3K9me3 (red) and DAPI (blue). Scale bars = 10 µm.
(3.65 MB TIF)

Text S1 Supplemental materials.
(0.05 MB DOC)
