# Supplementary figures and images for: Differential Localization and Independent Acquisition of the H3K9me2 and H3K9me3 Chromatin Modifications in the Caenorhabditis elegans Adult Germ Line
Source: PLoS Genet. 2010 Jan 22;6(1):e1000830. doi: 10.1371/journal.pgen.1000830 (PMC2809760; doi:10.1371/journal.pgen.1000830)

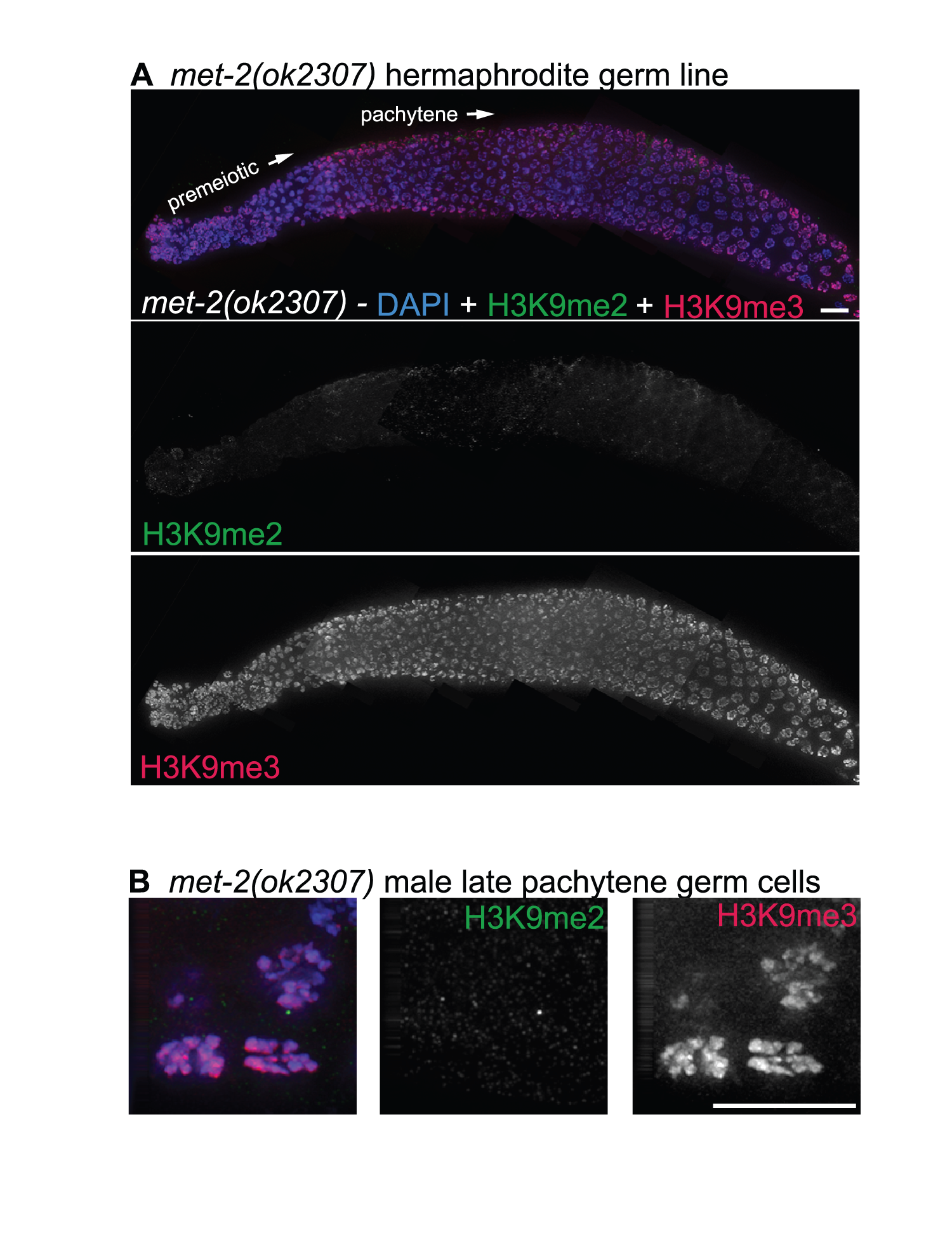

Supplement: Figure S1 — met-2(ok2307) is required for H3K9me2 in the adult germ line. (A) A germ line dissected from a met-2(ok2307) C. elegans hermaphrodite. The premeiotic region of the germ line is on the left, while germ cells at the diplotene stage of meiotic prophase are on the right. Top: anti-H3K9me2 staining (green) and anti-H3K9me3 staining (red) overlaid on DAPI-stained chromosomes (blue). Middle: anti-H3K9me2 staining alone. Bottom: anti-H3K9me3 staining alone. No chromosomal H3K9me2 staining is visible in the met-2(ok2307) mutant germ line. (B) met-2(ok2307) male pachytene germ cell nuclei, stained with anti-H3K9me2 and anti-H3K9me3 antibodies. In the pachytene nuclei from the met-2(ok2307) mutant male, H3K9me2 is no longer detected on the unpaired male X chromosome, while the distribution of H3K9me3 is unaffected. Blue = DAPI, Green = H3K9me2, Red = H3K9me3. Scale bar = 10 µm. (1.61 MB TIF) [file pgen.1000830.s001.tif]

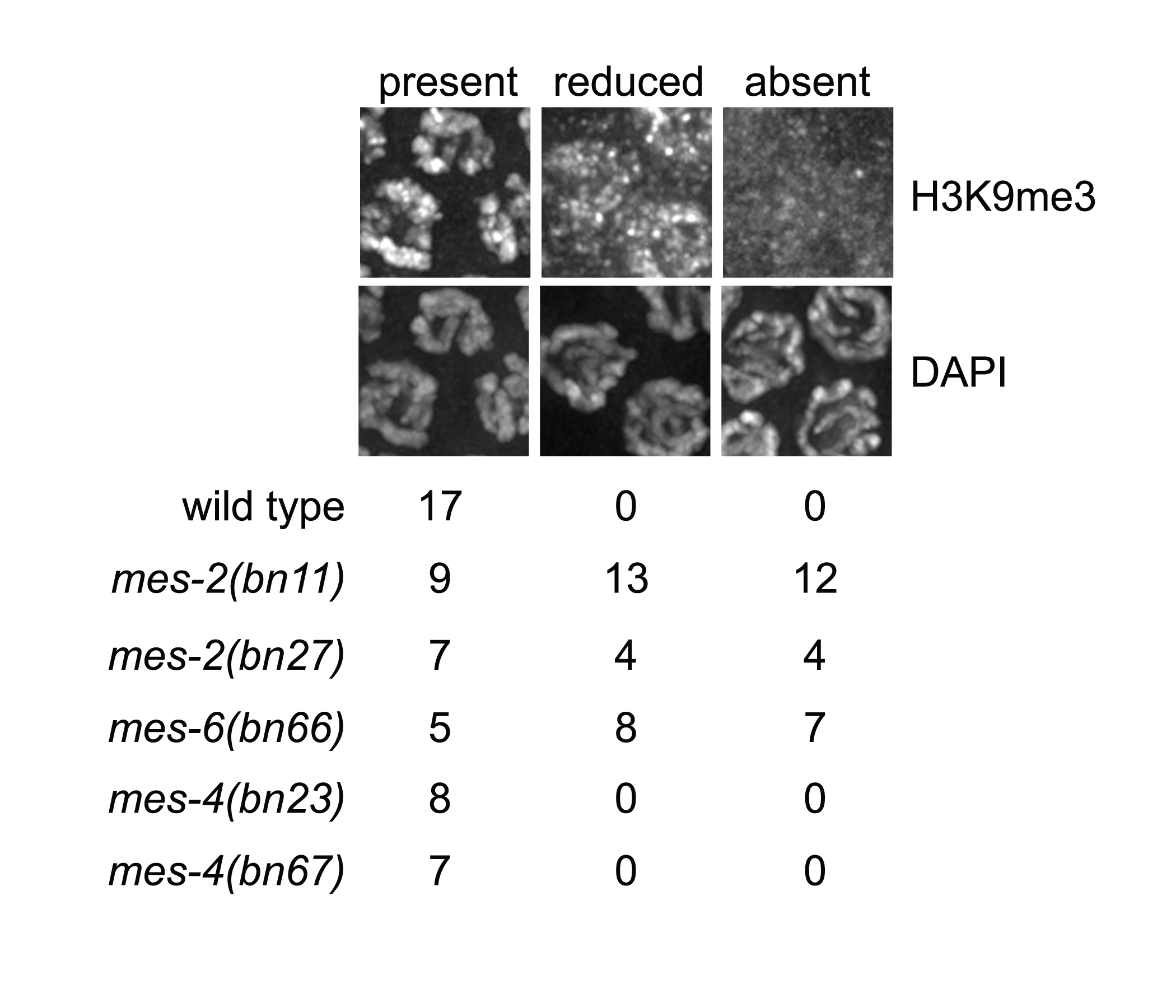

Supplement: Figure S2 — Status of H3K9me3 in the germ cells of mes mutants. Wild type, mes-2(bn11), mes-2(bn27), mes-4(bn23), mes-4(bn67) and mes-6(bn66) germ lines categorized with respect to H3K9me3 staining of germ cell nuclei from the premeiotic region through to the end of the pachytene stage. Panels at the top of the figure depict pachytene nuclei representing each of the three staining categories used. Germ line staining was categorized as “present” when the H3K9me3 staining was almost indistinguishable from wild type H3K9me3 staining. Germ line staining was classified as “reduced” in cases where the H3K9me3 signal was clearly fainter and readily distinguishable from that seen in wild-type germ lines, but individual nuclei could still be discerned based on anti-H3K9me3 staining. Germ line staining was classified as “absent” when chromosomal H3K9me3 was indistinguishable from background levels. (0.78 MB TIF) [file pgen.1000830.s002.tif]

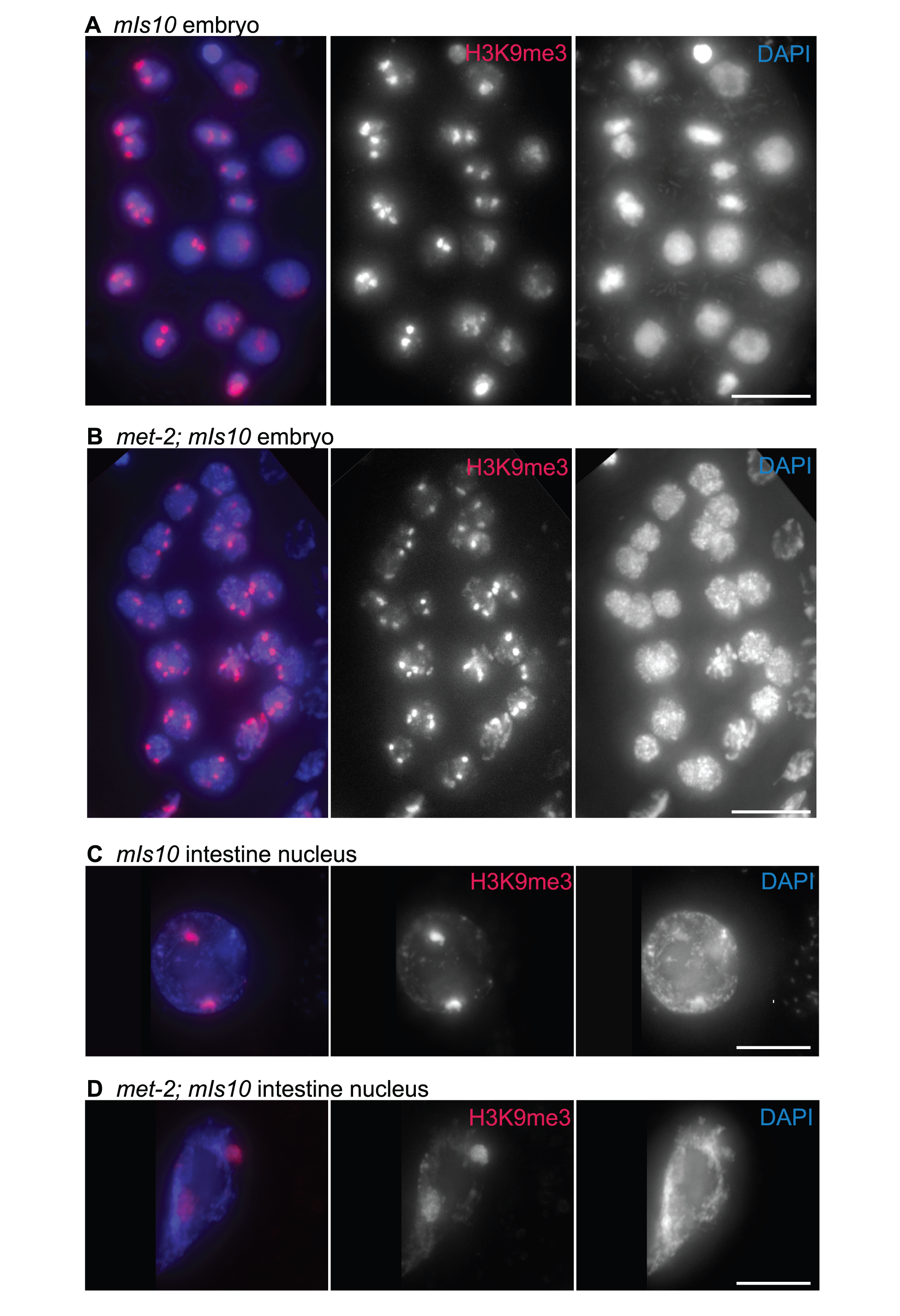

Supplement: Figure S3 — H3K9me3 marks mIs10 in embryos and adult intestine. (A) A mIs10∼16 cell embryo. Bright regions of mIs10-dependent H3K9me3 staining are apparent in all embryonic cells. (B) A met-2; mIs10∼22 cell embryo. In the met-2; mIs10 embryo, mIs10-dependent H3K9me3 staining is still detected. (Nuclei at the far right of the images are not part of the embryo). (C) A polyploid intestinal nucleus from an adult mIs10 hermaphrodite. Two regions enriched for H3K9me3 staining are present, corresponding to the presence of the mIs10 array. (D) A polyploid intestinal nucleus from an adult met-2; mIs10 hermaphrodite; two mIs10-dependent H3K9me3 signals are still detected. Embryos and intestinal nuclei have been co-stained with anti-H3K9me3 (red) and DAPI (blue). Scale bars = 10 µm. (3.65 MB TIF) [file pgen.1000830.s003.tif]
