# Supplementary material for: Differential Localization and Independent Acquisition of the H3K9me2 and H3K9me3 Chromatin Modifications in the Caenorhabditis elegans Adult Germ Line
Source: PLoS Genet. 2010 Jan 22;6(1):e1000830. doi: 10.1371/journal.pgen.1000830 (PMC2809760; doi:10.1371/journal.pgen.1000830)
Supplement: Text S1 — Supplemental materials. (0.05 MB DOC) [file pgen.1000830.s004.doc]

**Supplemental Text for Bessler *et al.*:**

**Supplemental Methods:**

**Immunofluorescence:** 18-20 hours post-L4 worms were dissected on a slide in egg buffer (250 mM HEPES, 1.18 M NaCl, 480 mM KCl, 20 mM CaCl_2_ and 20 mM MgCl_2_) + 0.1% Tween-20. 2.0% paraformaldehyde is added for fixation (5 minutes). Samples were freeze cracked by immersing slides in liquid nitrogen; slides were then placed into ice cold 100% methanol for 1 minute. Slides were washed for 3X 5 minutes in PBS + 0.1% Tween-20. Slides were then placed in blocking solution (PBS + 0.1% Tween-20 with 0.5% BSA) for 1 hour. Slides were dried without letting the specimen dry out and then primary antibody was added and incubated overnight at room temperature. The following morning, slides were washed 3X 10 minutes in PBS + 0.1% Tween-20 and secondary antibody was added. The slides were then incubated at room temperature, in the dark, for 2 hours. Slides were again washed 3X 10 minutes in PBS + 0.1% Tween-20. 50 μl 2 μg/ml DAPI was placed on slide and which was then incubated for 10 minutes at room temperature in the dark. The slides were washed again and vectashield (Vector Laboratories) was placed onto the specimen and slides were sealed for analysis.

**Whole Worm Ethanol Fixation:** A procedure modified from [1]. Worms were picked directly into a minimal volume of M9 on a microscope slide and excess liquid was wicked away. Whole worms were fixed by adding 15 μl of 95% ethanol. Once dry, ethanol was added to the worms twice more. A 1:1 mixture of DAPI:Vectashield (Vector Laboratories) was then added to the worms and the slides were sealed. Slides were stored up to 4 days at 4° before analysis.

**IF/FISH:** IF was performed as above, except that after washing off the secondary antibody the sample was fixed again in 3.7% paraformaldehyde for 15 minutes. The slides were washed first in PBS + 0.1% Tween-20, followed by two washes in 2X SSC + 0.1% Tween-20. Slides were next placed in 50% formamide/50% 2X SSC + 0.1% Tween-20 at 37°C for 1 hour. Excess solution was removed and 2 μl of probe, diluted in 13 μl hybridization buffer (50% formamide/50% 2X SSC + 0.1% Tween-20 with 10% w/v dextran sulfate), was added to the specimen. The slides were incubated overnight at 37°C using an Omnislide thermocycler (Thermo). The following morning slides were washed 2X 15 minute in 50% formamide/50% 2X SSC + 0.1% Tween-20 at 37°C, followed by 3X 10 minute washes in 2X SSC + 0.1% Tween-20 at room temperature. 50 μl 2 μg/ml DAPI was placed on slide and incubated for 10 minutes at room temperature in the dark. The slides were washed again and vectashield (Vector) was placed onto the specimen and slides were sealed for analysis.

**Supplemental Reference:**

1. Pepper AS, Killian DJ, Hubbard EJ (2003) Genetic analysis of Caenorhabditis elegans glp-1 mutants suggests receptor interaction or competition. Genetics 163: 115-132.
